# Integration of bioinformatics and machine learning approaches for the validation of pyrimidine metabolism-related genes and their implications in immunotherapy for osteoporosis

**DOI:** 10.1186/s12891-024-07512-z

**Published:** 2024-05-22

**Authors:** Zichen Feng, Zixuan Wu, Yongchen Zhang

**Affiliations:** 1 Shandong University of Traditional Chinese Medicine, Jinan, China; 2 Guangzhou University of Chinese Medicine, Guangzhou, Guangdong Province 510006 China

**Keywords:** Osteoporosis (OP), Pyrimidine metabolism genes (PyMGs), DEGs, WGCNA, Bioinformatics

## Abstract

**Background:**

Osteoporosis (OP), the “silent epidemic” of our century, poses a significant challenge to public health, predominantly affecting postmenopausal women and the elderly. It evolves from mild symptoms to pronounced severity, stabilizing eventually. Unique among OP’s characteristics is the altered metabolic profile of affected cells, particularly in pyrimidine metabolism (PyM), a crucial pathway for nucleotide turnover and pyrimidine decomposition. While metabolic adaptation is acknowledged as a therapeutic target in various diseases, the specific role of PyM genes (PyMGs) in OP’s molecular response remains to be clarified.

**Methods:**

In pursuit of elucidating and authenticating PyMGs relevant to OP, we embarked on a comprehensive bioinformatics exploration. This entailed the integration of Weighted Gene Co-expression Network Analysis (WGCNA) with a curated list of 37 candidate PyMGs, followed by the examination of their biological functions and pathways via Gene Set Variation Analysis (GSVA). The Least Absolute Shrinkage and Selection Operator (LASSO) technique was harnessed to identify crucial hub genes. We evaluated the diagnostic prowess of five PyMGs in OP detection and explored their correlation with OP’s clinical traits, further validating their expression profiles through independent datasets (GSE2208, GSE7158, GSE56815, and GSE35956).

**Results:**

Our analytical rigor unveiled five PyMGs—*IGKC, TMEM187, RPS11, IGLL3P,* and *GOLGA8N*—with significant ties to OP. A deeper dive into their biological functions highlighted their roles in estrogen response modulation, cytosolic calcium ion concentration regulation, and GABAergic synaptic transmission. Remarkably, these PyMGs emerged as potent diagnostic biomarkers for OP, distinguishing affected individuals with substantial accuracy.

**Conclusions:**

This investigation brings to light five PyMGs intricately associated with OP, heralding new avenues for biomarker discovery and providing insights into its pathophysiological underpinnings. These findings not only deepen our comprehension of OP’s complexity but also herald the advent of more refined diagnostic and therapeutic modalities.

**Supplementary Information:**

The online version contains supplementary material available at 10.1186/s12891-024-07512-z.

## Introduction

In 1993, the World Health Organization (WHO) recognized osteoporosis as a systemic skeletal disorder characterized by diminished bone mass, compromised bone tissue microarchitecture, increased fragility, and a heightened risk of fractures [1]. Termed the ‘silent epidemic of the twenty-first century,’ osteoporosis has become a paramount public health concern, representing the most common metabolic bone disorder [2]. It is chronic, progressively severe, and typically silent until the occurrence of the first fracture, placing it fourth among chronic diseases after cardiovascular diseases, dementia, and lung cancer, and highlighting its profound societal and economic impact [3, 4]. This asymptomatic nature often leads to delayed diagnoses, emphasizing the urgent need for the early detection and intervention biomarkers [5]. Osteoporosis’s complex etiology involves lifestyle choices, disease conditions, environmental exposures, and hormonal dynamics, each playing a crucial role in its risk and progression [6]. Excessive alcohol consumption directly impairs osteoblast function, disrupts calcium balance, and induces oxidative stress, all of which contribute to bone degradation and an elevated risk of osteoporosis [7]. Similarly, autoimmune diseases like rheumatoid arthritis and systemic lupus erythematosus exacerbate osteoporosis through chronic inflammation and the autoimmune destruction of bone tissue. Intriguingly, viral infections, notably hepatitis B virus (HBV), are linked to osteoporosis via mechanisms such as liver dysfunction affecting vitamin D and bone-related hormone metabolism and the chronic inflammation leading to bone loss [8]. Hormonal imbalances, including deficiencies in estrogen, testosterone, and thyroid hormones, are pivotal in osteoporosis development, with estrogen deficiency in postmenopausal women notably accelerating bone turnover and density loss [9]. Furthermore, environmental pollutants, including heavy metals and endocrine-disrupting chemicals, pose significant threats to bone health by mimicking or inhibiting hormonal actions essential for bone maintenance or directly harming bone cells [10]. In conclusion, the interplay between osteoporosis and factors like alcohol consumption, autoimmune diseases, viral infections (especially HBV), hormonal imbalances, and environmental pollutants is intricate, highlighting the necessity for comprehensive prevention, diagnosis, and treatment strategies.

In oncology, the identification of metabolic reprogramming as a hallmark of cancer marks a significant shift, recognizing its pivotal role in supporting tumor proliferation and survival [11]. This transformation results in a unique metabolic phenotype within tumor cells, leading to profound changes in the tumor microenvironment (TME) [12]. Characterized by a diverse cellular composition and a complex extracellular matrix, the TME often suffers from poor oxygen and nutrient levels due to inadequate vascularization [13]. Recent studies have brought to light the significance of non-tumor immune infiltration within the TME, highlighting a critical link between immune responses and metabolic alterations, including nutrient depletion, elevated oxygen consumption, and reactive species production [14]. This interaction suggests that metabolic modulation in the TME could potentially amplify the efficacy of immunotherapies, indicating a promising avenue for cancer treatment enhancement through targeted metabolic strategies [15]. Pyrimidine metabolism (PyM) encompasses essential biochemical pathways for the synthesis, degradation, and utilization of pyrimidine nucleobases, such as cytosine and uracil, critical for DNA and RNA composition. PyM plays a crucial role in maintaining nucleic acid synthesis balance and overall energy metabolism, involving pathways for pyrimidine compound production and breakdown [16]. This includes the orchestrated activities of enzymes like dihydroorotate dehydrogenase (DHODH), uridine monophosphate synthase (UMPS), and orotidine 5'-phosphate decarboxylase (OMPDC), central to the synthesis of uridine monophosphate (UMP) [17]. The heightened demand for pyrimidine nucleotides in rapidly dividing cells is predominantly met through de novo synthesis, whereas differentiated cells rely on salvage pathways [18]. The exploration of PyM offers new therapeutic prospects, including purinosome formation and PyMG therapies, yet the connection between PyM and immunotherapy, particularly in OP, remains an area ripe for exploration. This study aims to investigate PyMGs and their relationship with immunotherapy in OP, striving to enhance our understanding and uncover novel therapeutic opportunities.

**Fig. 1:**
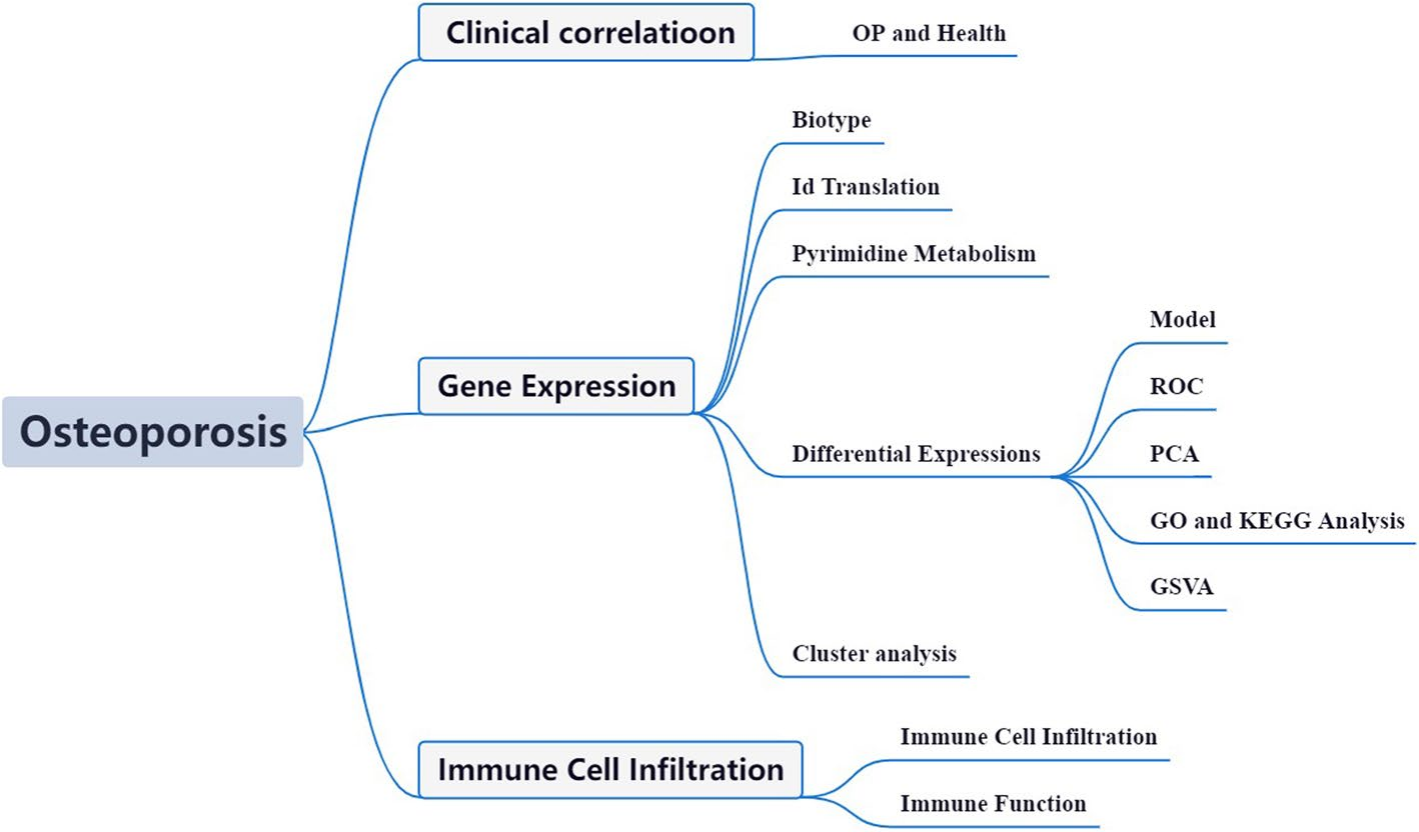
Framework

The emergence of high-throughput transcriptome sequencing, coupled with the comprehensive clinical datasets from the OP Initiative, heralds a new era for dissecting the intricate transcriptional dynamics and molecular pathways underlying OP. Utilizing these data-rich resources, bioinformatics analyses have unveiled profound insights, substantially advancing our understanding of the pathophysiology and molecular basis of OP from varied perspectives [19, 20]. Despite these strides, the role of PyMGs in OP represents a largely unexplored territory within bioinformatics research. Consequently, this study embarked on an investigation into OP-related datasets from the GEO, with a particular emphasis on PyMGs (Fig. 1). This initiative seeks to fill the existing knowledge void, providing a fresh lens through which to view OP at the molecular scale.

## Materials and methods

We used the approaches proposed by Zi-Xuan Wu, et al. 2023 [21].

### Data acquisition

For this study, we harvested raw data from the GEO series, encompassing GSE2208, GSE7158, GSE56815, and GSE35956, utilizing the GPL96 and GPL570 platforms. Datasets GSE2208, GSE7158, and GSE56815 were designated for the training phase, while GSE35956 was reserved for testing purposes (Table 1). A curated list of 105 PyMGs was sourced from the MSigDB, as delineated in Supplementary Table S1.

**Table 1:** The clinical characteristics of patients

Variables	Number of samples			
	GSE2208	GSE7158	GSE56815	GSE35956
Gender				
Male/Female	unknown	unknown	0/80	1/9
Diagnosis				
high BMD/low BMD	10/9	14/12	40/40	5/5

### Differential expression analysis

We employed Perl scripts for the processing and alignment of mRNA data. After standardizing data from the training datasets, differential expression analysis was conducted, employing a threshold of False Discovery Rate (FDR) < 0.05 and an absolute log2 fold change (|log2FC|) ≥ 1, to discern variations in PyMG expression. This led to the identification of differentially expressed PyMGs (DEGs). Complementarily, the support vector machine recursive feature elimination (SVM-RFE) technique, facilitated by the e1071 package, was employed to devise an advanced machine learning model. Support vector machines (SVMs) are a category of robust, generalized linear classifiers within the supervised learning sphere, adept at executing binary classification tasks with high precision. SVMs uniquely incorporate the hinge loss function for empirical risk assessment, while the integration of regularization terms in their algorithm enhances sparsity and resilience, thereby optimizing structural risk. The application of kernel methods enables SVMs to transcend linear constraints, establishing them as leading figures in kernel learning techniques.

### Immune cell infiltration and clustering

Immune cell composition within the samples was analyzed using the CIBERSORT algorithm. Subsequently, prognostic-related PyMGs were stratified into two distinct clusters, labeled as Cluster 1 and Cluster 2, based on their expression profiles.

### Functional enrichment analysis

To elucidate the biological functions and pathways associated with the identified PyMGs, Gene Ontology (GO) and Kyoto Encyclopedia of Genes and Genomes (KEGG) analyses were employed. Using R programming, we assessed the impact of these differentially expressed PyMGs on biological processes, molecular functions, and cellular components. Gene Set Variation Analysis (GSVA) was subsequently applied to further delineate these interactions.

### Co-expression gene network construction

WGCNA was utilized to categorize genes and investigate their associations with OP characteristics. The network was constructed from genes exhibiting the top 25% variance in datasets GSE2208, GSE7158, and GSE56815. Module identification within this network was performed using the dynamic tree cutting method with a defined threshold of 0.25.

### PyMGs identification and validation

Intersection analysis was conducted to identify PyMGs, encompassing DEGs from pivotal modules (identified via WGCNA), PyM, and cluster hub genes. These overlapping genes were visualized through a Venn diagram. Enrichment pathways and biological mechanisms were further examined. Following the identification of hub PyMGs, datasets GSE2208, GSE7158, and GSE56815 were segmented into training cohorts, and external validation was performed using dataset GSE35956. Prognostic assessments for the test group were correlated with clinical data, particularly age.

### Drug-gene interaction analysis

In the current bioinformatics-driven era, the pursuit of effective disease biomarkers for diagnosis has intensified. Beyond their identification, these biomarkers’ clinical application in predictive drug response analytics is crucial for advancing Osteoporosis prevention and treatment. Validated biomarkers act as guiding lights for targeted therapies, making accurate drug-gene interaction predictions essential. In this research, we employed the Drug-Gene Interaction database DGIdb (https://dgidb.genome.wustl.edu/) to predict potential drug interactions with our identified hub genes.

## Results

### Differential expression of PyMGs and principal component analysis

**Fig. 2:**
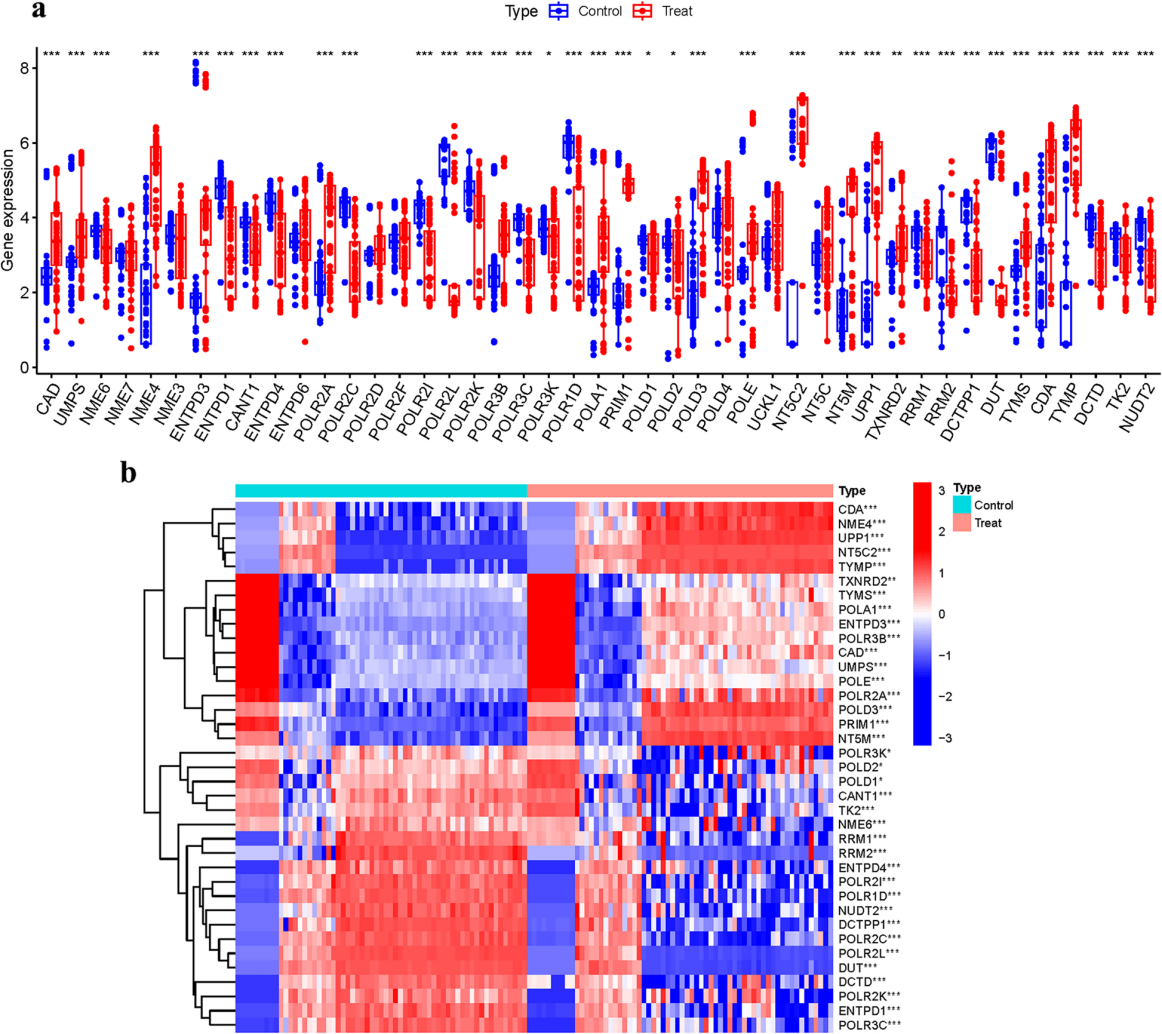
Principal component analysis. **a** Gene expression. **b** Heatmap

Our analysis revealed significant differential expression in 21 out of 37 PyMGs including *CAD, UMPS, NME6,* and *NME7* (Fig. 2a). Distinct clustering patterns were observed among these genes, with some preferentially expressed in the treatment group (e.g., *CDA, NME4, UPP1*) and others in the control group (e.g., *RRM2, ENTPD4, POLR2I*) (Fig. 2b, Supplementary Table S2).

### Chromosomal localization and correlation analysis of PyMGs

**Fig. 3:**
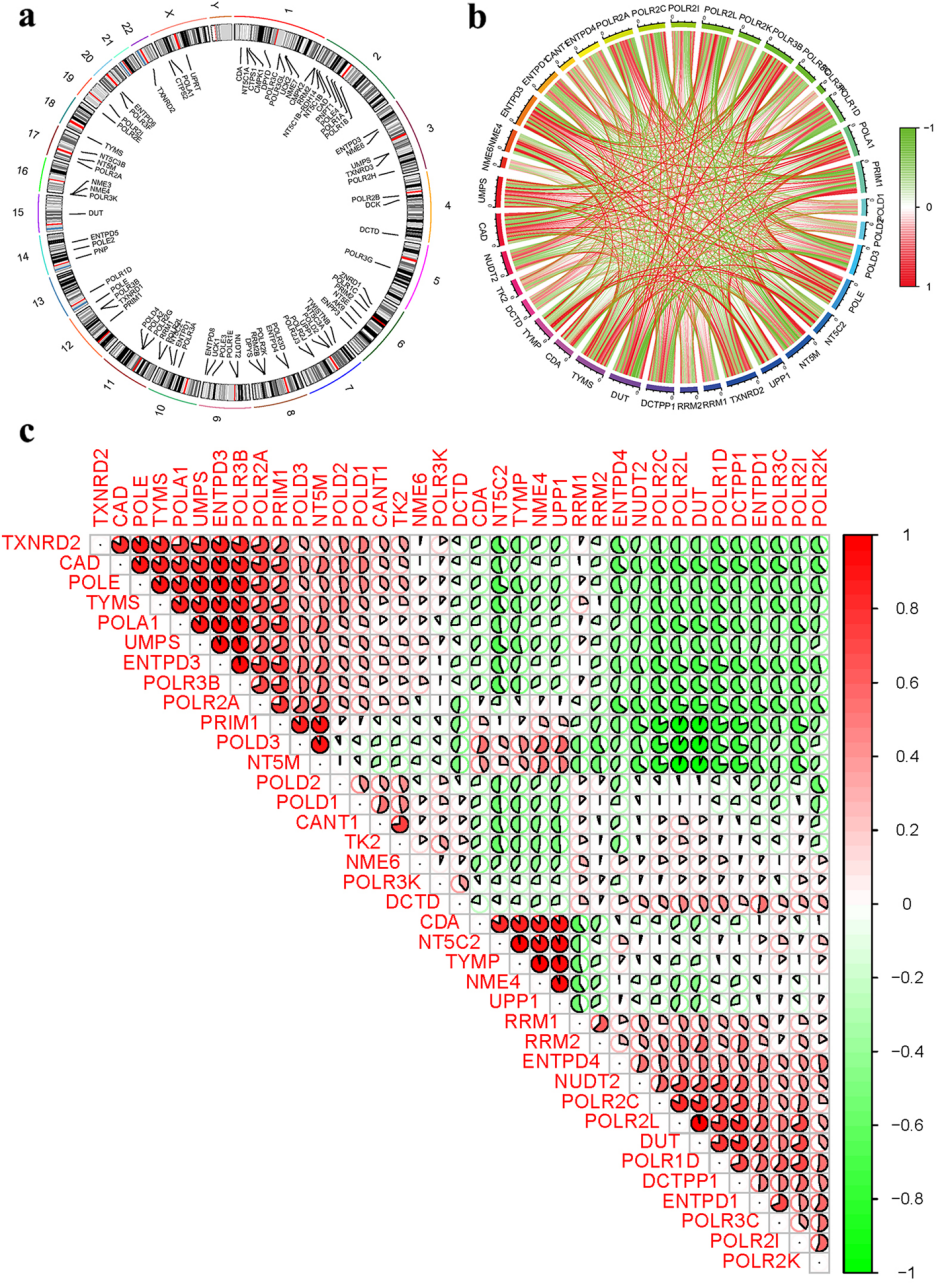
PyMGs. **a** PyMGs on sequences. **b**, **c** PyMGs and related genes

We used perl software to calculate PyMG and then visualized it through R’s RCircos package. The chromosomal positions of PyMGs were mapped and visualized (Fig. 3a, Supplementary Table S3). Corrplot and circlize packages were used to visualize the relationship between PyMGs according to their expression. Subsequent correlation analyses further detailed the interrelationships between these genes. Predictably, these genes are closely related to each other (Figs. 3b, c).

### Analysis of immune cell infiltration

**Fig. 4:**
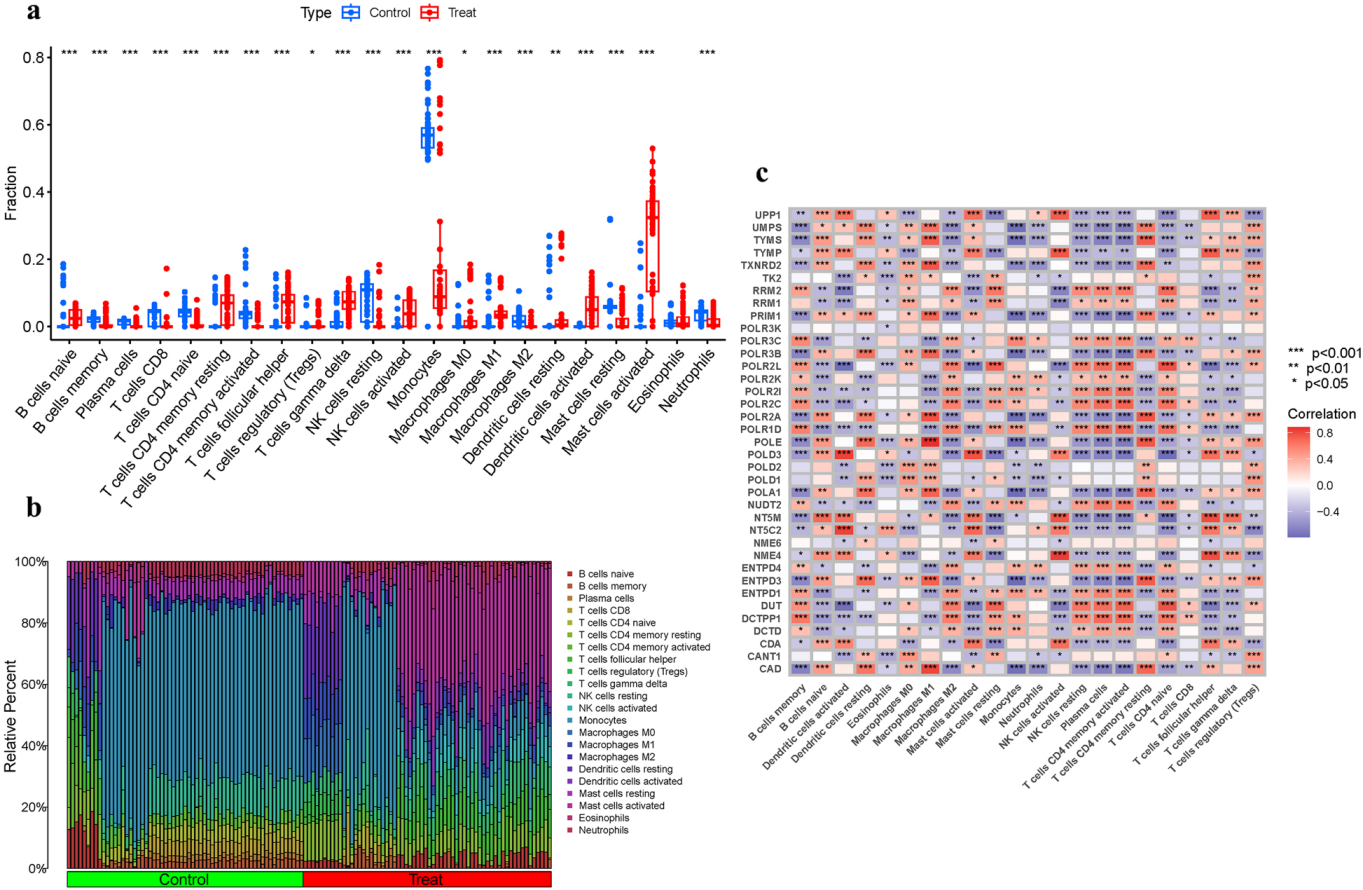
Expression of Immune cells. **a**, **b** Expression of immune cells in different clusters. **c** Correlation between PyMGs and immune cells

Considering the pivotal role of the immune milieu in OP pathogenesis, we depicted immune cell distributions using bar and correlation plots (Figs. 4a-c), highlighting the intricate relationships between immune cells and PyMG expression.

### PyMG-driven clustering in OP

**Fig. 5:**
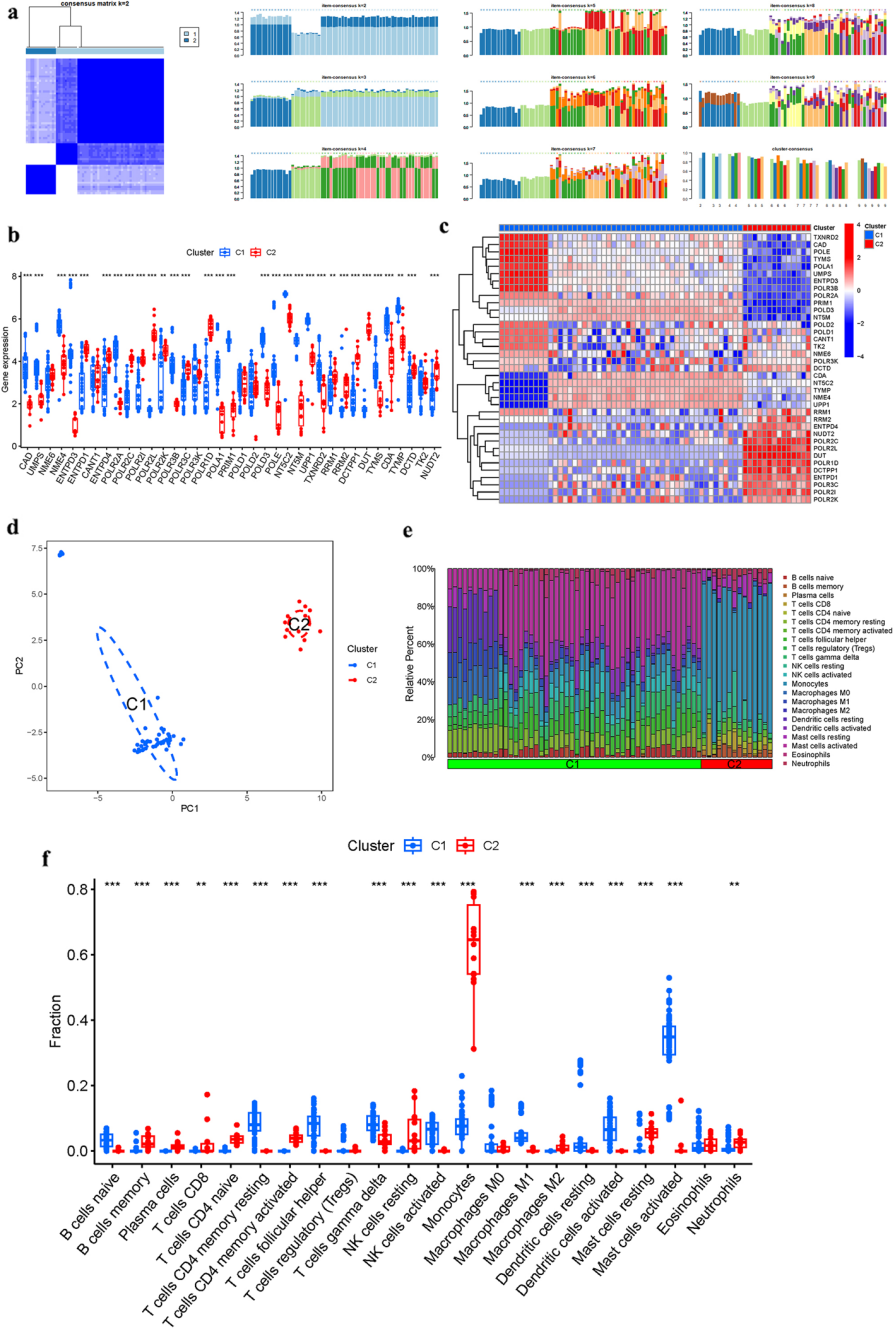
Cluster analysis. **a** Consensus. **b**, **c** PyMGs in clusters. **d** PCA. **e**, **f** Immune cell infiltration

Cluster analysis with k = 2 yielded the highest intragroup correlations, suggesting that PyMGs can stratify OP patients into two distinct groups (Fig. 5a). Analysis of PyMG expression in these clusters showed no significant difference in *NME6, CANT1, POLR3K, POLD1, POLD2*, and *TK2* levels between the groups (Figs. 5b, c). PCA further segregated patients into different risk categories (Fig. 5d), and immune cell infiltration patterns were also examined in this context (Figs. 5e, f).

### Functional enrichment analysis

**Fig. 6:**
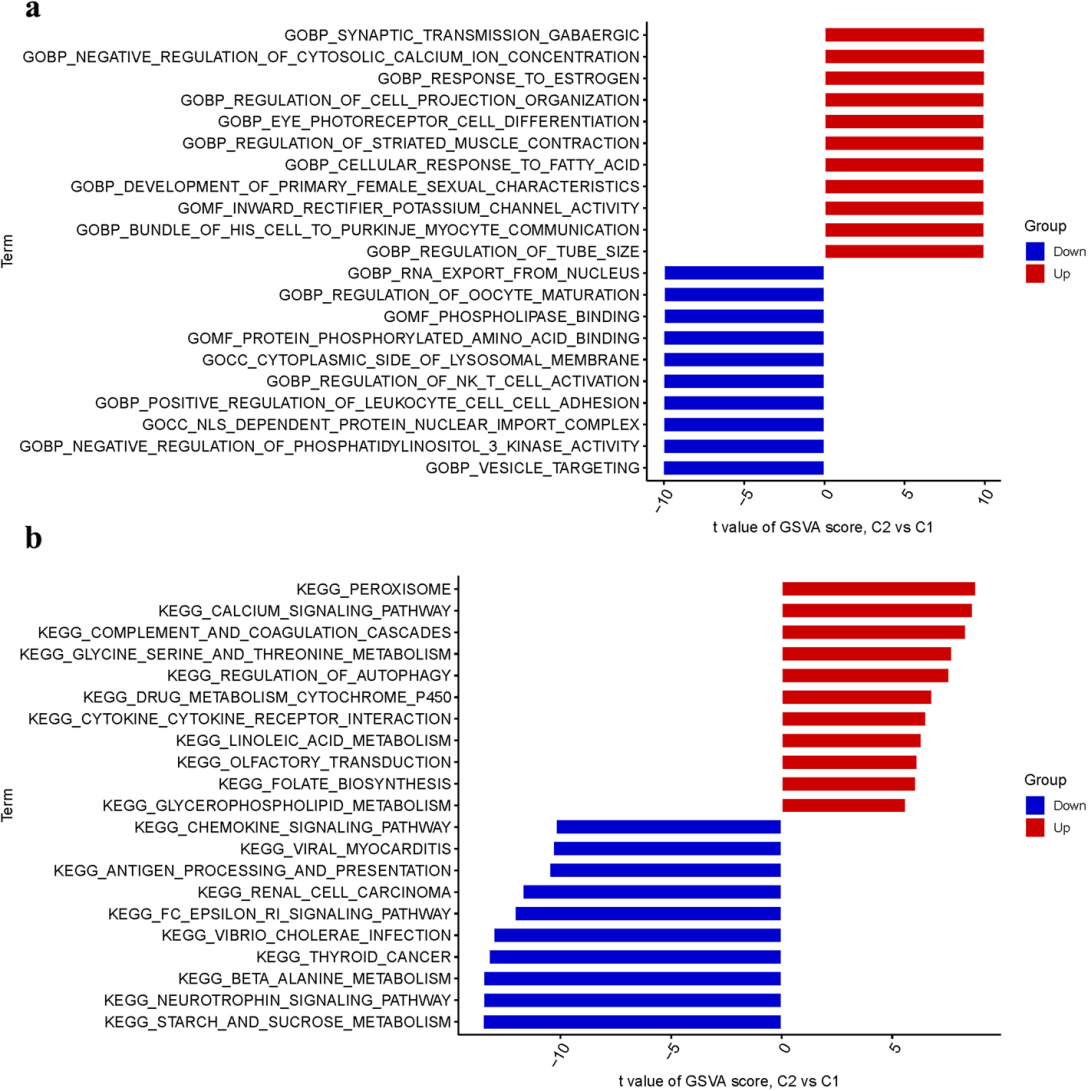
Enrichment analysis for DEGs. **a** GO. **b** KEGG

Functional enrichment of PyMGs revealed MF primarily related to inward rectifier potassium channel activity and phospholipase binding. BP predominantly involved the regulation of cytosolic calcium ion concentration and GABAergic synaptic transmission, while CC were primarily associated with the lysosomal membrane and nuclear import complex (Fig. 6a). Pathway analysis highlighted enrichment in peroxisome, calcium signaling, and complement/coagulation cascades (Fig. 6b).

### Co-expression network construction

**Fig. 7:**
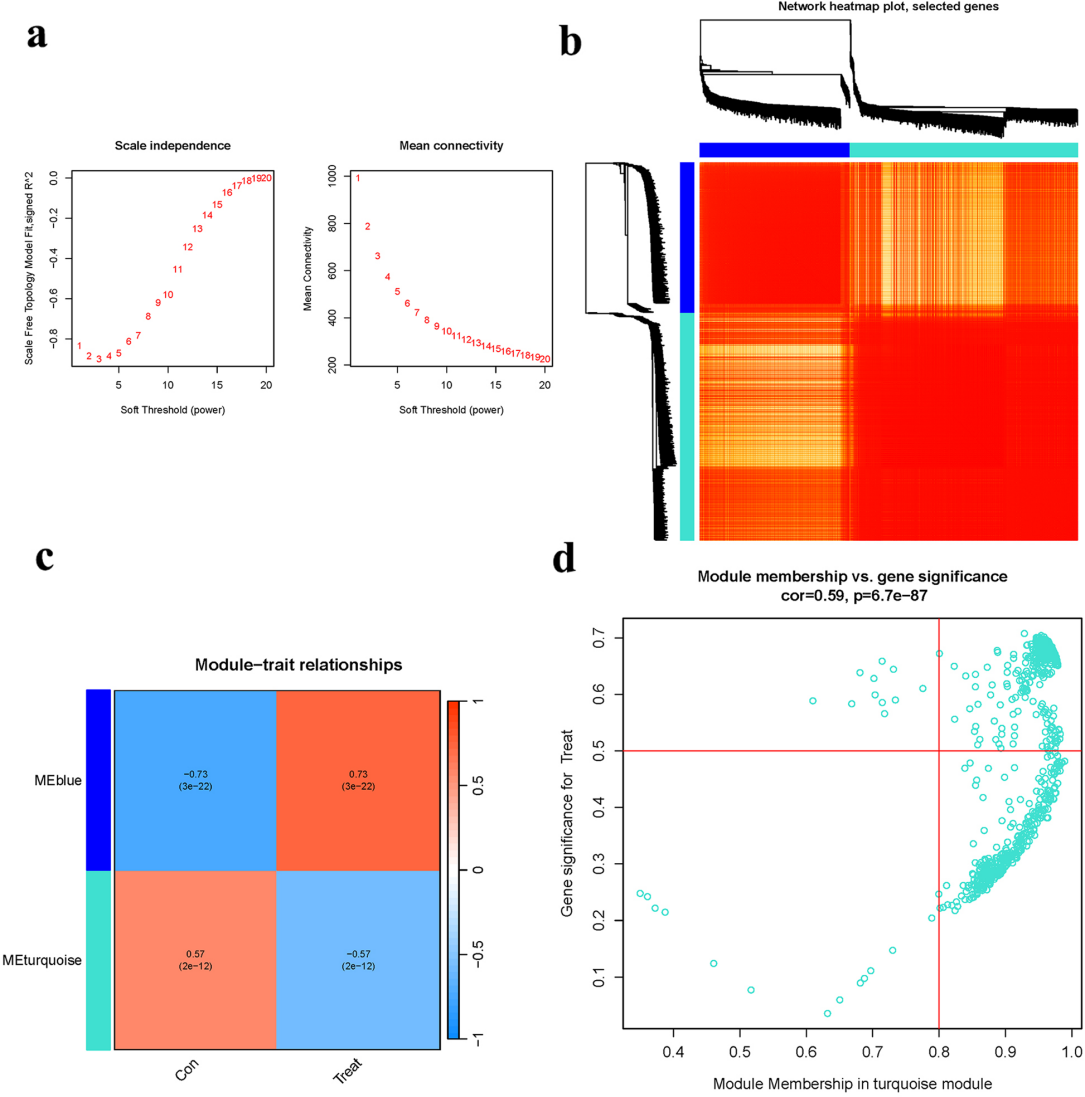
Co-expression module. **a** Index analysis. **b** Dendrogram clustering. **c** Heatmap. **d** Scatterplot

We employed a soft-thresholding power to approximate a scale-free topology for the gene network (Fig. 7a). The most variable genes were integrated into two co-expression modules (Fig. 7b), with Pearson’s correlation analysis further elucidating these relationships (Fig. 7c). The turquoise module exhibited a strong connection with the group attribute (OP and Control) (Fig. 7d).

### Clustering within the co-expression network

**Fig. 8:**
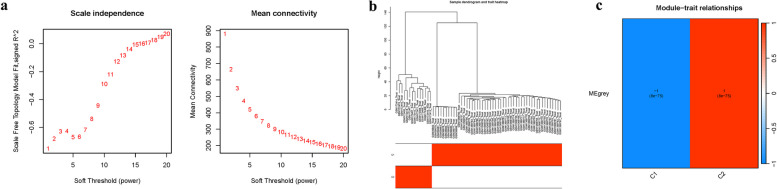
Cluster co-expression modules **a** Iindex analysis. **b** Dendrogram clustering **c** Heatmap

An additional network was constructed (Fig. 8a), with clustering of variance genes leading to distinct co-expression modules (Fig. 8b). We explored the relationship between module eigengenes and clinical features, finding the grey module strongly associated with the group characteristic (Fig. 8c).

### Model construction

**Fig. 9:**
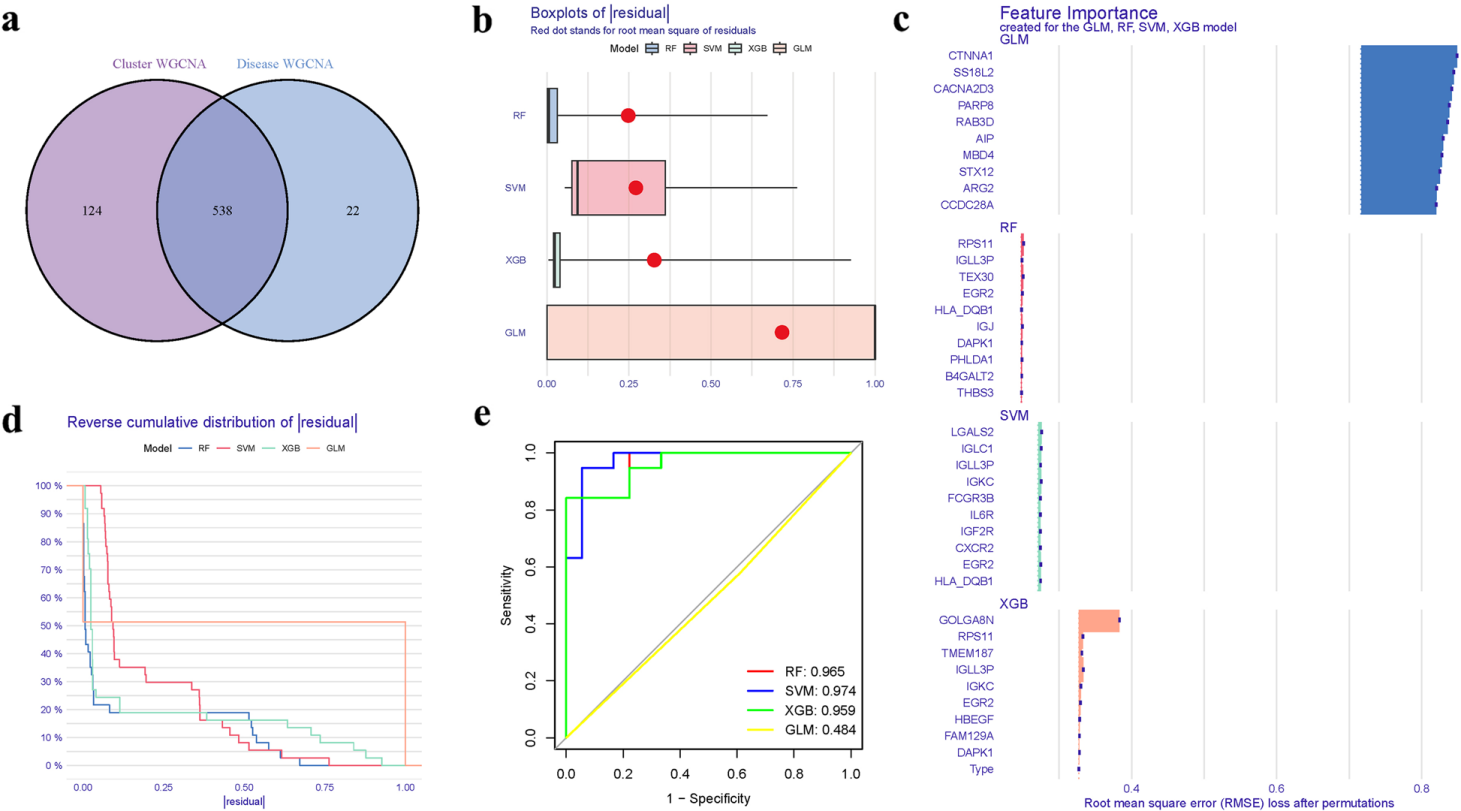
Model. **a** Venn. **b**, **c** Residual expression patterns. **d** Model trend chart. **e** AUC of train group

Intersecting DEGs, turquoise module genes, and PyMGs identified key overlapping genes (Fig. 9a, Supplementary Table S5). Boxplots represented the expression patterns of these genes in OP (Fig. 9b). Subtle differences were observed in the proportions of the four models (Fig. 9c), with varying predictive values across different stages (Fig. 9d). The diagnostic potential of PyMGs in distinguishing OP from control samples showed promising results, with AUCs for RF: 0.965, SVM: 0.974, XGB: 0.959, GLM: 0.484 (Fig. 9e), with the XGB model being the most precise.

### Model validation

**Fig. 10:**
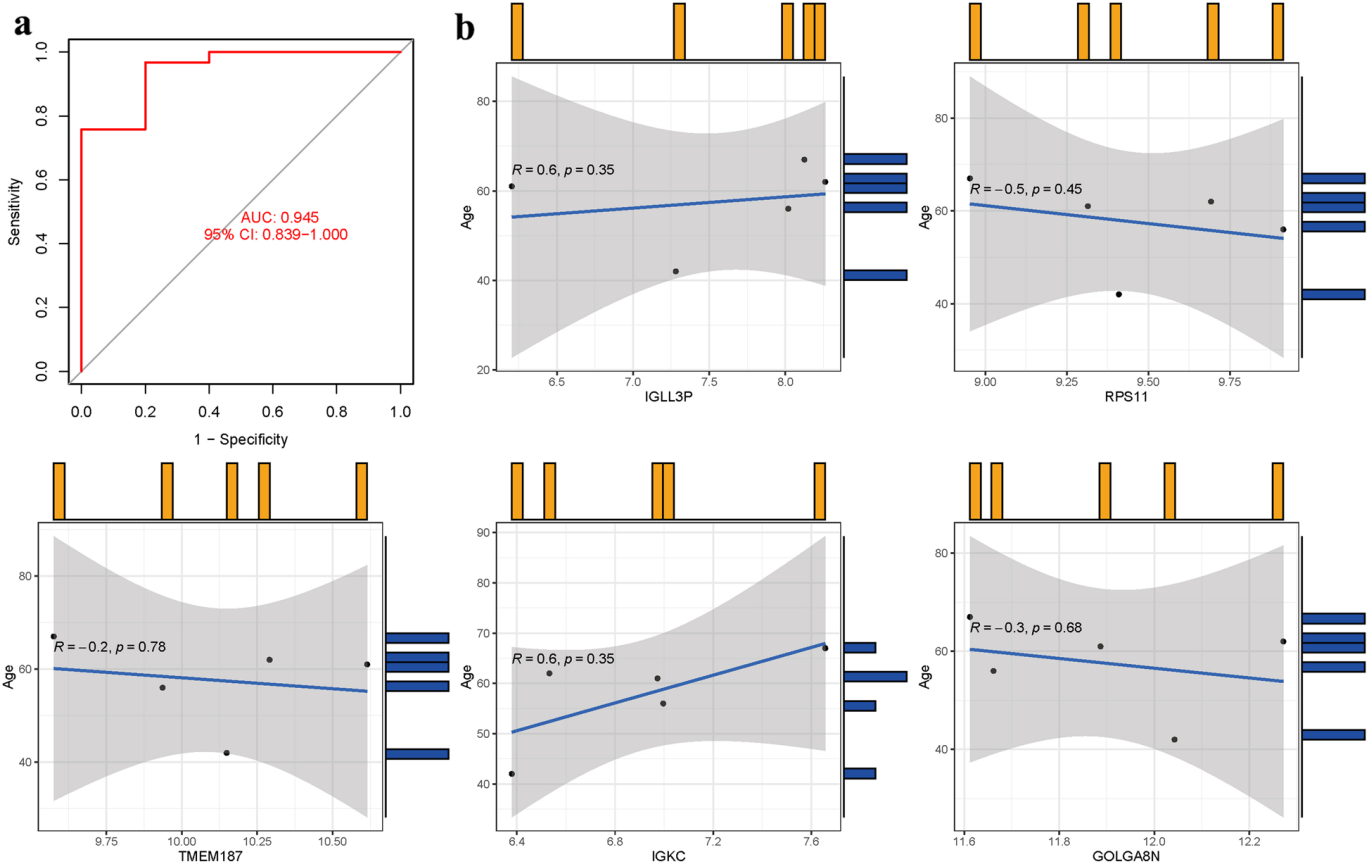
**a** AUC of test group. **b** Analysis of the relationship between hub genes and age

The model validation in dataset GSE35956 yielded an AUC of 0.945 (95% CI 0.839–1.000) (Fig. 10a). The correlations between five hub genes and age were also analyzed, revealing mixed associations, although the *p*-values were all above 0.05 (Fig. 10b, Supplementary Table S6).

#### Drug-gene interaction analysis

We utilized all interacting genes for drug prediction, which encompassed 334 candidates including GEMCITABINE, SORAFENIB, DASATINIB, among others (Table 2 and Supplementary Table S7).

**Table 2:** Gene related drugs

Gene	Match_type	Drug	Interaction_types
CPT1B	Definite	PERHEXILINE	unknown
CPT2	Definite	PERHEXILINE	inhibitor
HSD17B10	Definite	CHEMBL72365	unknown
HSD17B10	Definite	CARBIDOPA	unknown
HSD17B10	Definite	AURINTRICARBOXYLIC ACID	unknown
HSD17B10	Definite	CHEMBL516616	unknown
HSD17B10	Definite	MERCURIC CHLORIDE	unknown
HSD17B10	Definite	AMPHOTERICIN B	unknown
HSD17B10	Definite	N,N'-DIPHENYL-P-PHENYLENEDIAMINE	unknown
HSD17B10	Definite	SKIMMIANINE	unknown
HSD17B10	Definite	DIETHANOLAMINE	unknown
HSD17B10	Definite	RESOKAEMPFEROL	unknown
HSD17B10	Definite	CHEMBL259421	unknown
HSD17B10	Definite	2-AMINOANTHRACENE	unknown
HSD17B10	Definite	HAEMATOXYLIN	unknown
HSD17B10	Definite	4'-METHOXYFLAVONE	unknown
HSD17B10	Definite	CHEMBL602213	unknown
HSD17B10	Definite	THIMEROSAL	unknown
HSD17B10	Definite	ALRESTATIN	unknown
HSD17B10	Definite	THYROXINE	unknown
HSD17B10	Definite	CHEMBL296407	unknown
HSD17B10	Definite	METIZOLINE	unknown
HSD17B10	Definite	CHEMBL201325	unknown
HSD17B10	Definite	CHEMBL275260	unknown

## Discussions

OP is a pervasive systemic bone disease marked by reduced bone density and mass, compromised bone microarchitecture, increased fragility, and a higher risk of fractures [22]. It presents a significant health concern, affecting one in three women and one in five men over 50, with osteoporotic fractures often remaining undetected until occurrence, thus imposing considerable physical, personal, and economic burdens [23]. Beyond its role in glucose metabolism, proliferating cancer cells also extensively utilize Pyrimidine Metabolism (PyM) for energy and as a source of building blocks, with many demonstrating a critical dependence on exogenous pyrimidines for survival [24]. This dependency has shifted research focus towards non-tumor entities, underscoring the importance of exploring PyM’s wider implications. In cancer, cellular metabolism exhibits marked alterations, with enhanced glycolytic activity serving as a key physiological marker of malignancy [25]. Recent studies have illuminated the diagnostic and therapeutic relevance of metabolic markers like cysteine and nucleotide metabolism, alongside 2-hydroxyglutarate in gliomas, highlighting the pivotal role of gene expression regulation [26]. Investigations into PyM have sought to decode the complex relationship between metabolic dysregulation and genetic mutations in cancer [27]. Cells with mutations in KRAS, PTEN, or p53 show increased pyrimidine biosynthesis, rendering them vulnerable to synthetic lethality strategies that target this pathway, offering a promising therapeutic angle against tumors with gain-of-function mutations [28]. Furthermore, examining the intersections between the pyrimidine pathway and other metabolic routes offers insights into metabolic diversity, guiding personalized treatment approaches [29]. The advancements in cancer research have catalyzed a shift towards examining non-tumor elements to achieve a comprehensive understanding of non-cancer biology. In this vein, investigating distinct PyM patterns throughout OP’s progression presents substantial promise. Identifying varying PyM profiles as OP advances could deepen our grasp of PyM’s involvement in OP’s etiology, enabling the creation of precise therapeutic interventions.

In our investigation of OP, we identified thirty-seven DEGs associated with PyM. By integrating these DEGs with WGCNA and PyMGs, we underscored their potential significance in the pathogenesis of OP. Employing LASSO regression analysis, we pinpointed five pivotal PyMGs (*IGKC, TMEM187, RPS11, IGLL3P, GOLGA8N*), whose diagnostic value was further corroborated in independent datasets, thus emphasizing their importance within OP’s molecular framework. Nonetheless, our discoveries also lay the groundwork for future investigations, especially concerning the impact of these genes on specific transcription factors governing PyM regulation. Notably, IGKC, known for its role in immunological and cancer-related processes, along with TMEM187 and SYTL4—genes associated with autism spectrum disorders—show interactions with key genes in these areas. Their presence in the mRNA of extracellular vesicles within the nervous system highlights their importance in protein translation in target cells, marking them as potential research subjects in autism studies [30, 31]. These findings reinforce our conclusions on the role of PyM-related DEGs in OP pathogenesis. Furthermore, the study GSE35956 proposes a PyM-associated trait as a promising prognostic marker for OP, although exploration of gene alterations linked to PyM in this context is still in its early phases, suggesting ample scope for further exploration.

In the current field of OP research, genomics has emerged as a pivotal tool for elucidating the mechanisms underlying the disease and identifying potential therapeutic targets. Notably, specific genes such as *IGKC, TMEM187, RPS11, IGLL3P,* and *GOLGA8N* play crucial roles in the onset and progression of OP. The protein encoded by the *IGKC* gene is a vital component of the immune system, involved in antibody production. Against the backdrop of OP, the expression of *IGKC* may influence the bone marrow microenvironment and the interactions between osteoblasts and osteoclasts, thereby affecting the equilibrium of bone remodeling [32]. The *TMEM187* gene encodes a transmembrane protein that may participate in cell signaling pathways critical for the differentiation of osteoblasts and the activity of osteoclasts. Although the specific role of *TMEM187* in bone metabolism has not been fully elucidated, it suggests the potential of indirectly influencing the development of OP by modulating specific signaling pathways, such as the Wnt or BMP pathways [33]. *RPS11*, a ribosomal protein involved in protein synthesis, plays a central role in maintaining the functionality of bone cells and the health of bone tissue. Consequently, *RPS11* could indirectly contribute to the pathology of OP by regulating protein synthesis in osteoblasts and osteoclasts [34]. As a pseudogene, *IGLL3P* does not encode a protein but may participate in immune regulation and cell signaling by affecting the expression of related genes, thereby indirectly impacting the development of OP. *GOLGA8N*, a member of the golgin A8 family associated with the Golgi apparatus, may be involved in the transport and sorting mechanisms within cells [35]. Although the role of *GOLGA8N* in bone metabolism is yet to be thoroughly investigated, its function in intracellular transport suggests it might influence bone reconstruction by affecting the secretory activities of osteoblasts and the phagocytic abilities of osteoclasts [36]. In summary, these genes are linked to the development of OP through their unique mechanisms. Their functions likely influence the activities of osteoblasts and osteoclasts, mediate immune responses, and regulate intracellular signaling and protein synthesis. Future research should further explore the specific roles of these genes in OP and their potential as therapeutic targets for developing new treatment strategies. By gaining a deeper understanding of how these genes affect bone metabolism and the process of bone remodeling, we can move closer to more effectively preventing and treating OP.

The pyrimidine metabolic pathway comprises a series of enzyme-catalyzed reactions responsible for the synthesis and degradation of pyrimidine nucleotides, fundamental components of RNA and DNA. OP, characterized by reduced bone mass, deterioration of bone microarchitecture, increased skeletal fragility, and heightened risk of fractures, appears to be unrelated to pyrimidine metabolism at first glance [37]. However, recent research has illuminated the crucial role of pyrimidine metabolism in bone metabolism, particularly in the processes of bone formation and resorption. Initially, pyrimidine metabolites, such as uracil and thymine, play significant roles in the synthesis and degradation processes within bone cells, including osteoblasts and osteoclasts [38]. These metabolites are involved in signaling pathways, such as the Wnt/β-catenin pathway, which is pivotal for regulating bone formation and resorptive activities. For instance, uracil can promote the differentiation and maturation of osteoblasts, thereby enhancing bone formation [39]. Furthermore, abnormalities in pyrimidine metabolism are closely associated with the development of OP. Studies have shown that alterations in the activity of pyrimidine metabolizing enzymes can affect the functionality of bone cells, leading to bone loss. For example, mutations or decreased activity in specific metabolic enzymes are correlated with a heightened risk of OP [40]. Additionally, intermediates and products within the pyrimidine metabolic pathway, such as amino acids, nucleotides, and their derivatives, are essential for maintaining the overall health and functionality of bone tissue. They not only provide the energy and building blocks required for the proliferation and differentiation of bone cells but also regulate bone metabolism by activating or inhibiting specific signaling pathways [41]. In summary, the connection between pyrimidine metabolism and OP suggests that this metabolic pathway may serve as a potential therapeutic target for bone diseases. This relationship underscores the importance of further research into the specific roles of pyrimidine metabolism in bone health and the development of interventions to modulate this pathway for the prevention and treatment of OP.

Bone turnover, a critical physiological process entailing both bone formation and resorption, is intricately governed by a complex cytokine network [42]. This process is at the heart of the immunoskeletal interface, where extensive interactions between the immune system and bone cells occur, profoundly influencing bone turnover in both health and disease states. Notably, OP is often linked with chronic inflammatory conditions [43]. The decline in estrogen during menopause and aging-related changes are known to exacerbate OP by enhancing osteoclastogenic inflammatory cytokine production [44]. Inflammatory rheumatic diseases, which involve both local and systemic bone loss, serve as prime examples of the deep-seated interplay between the immune system and bone health, often leading to osteoclast hyperactivation and a disrupted balance in bone dynamics [45, 46]. In our preceding research, we delved into the expression patterns of PyMGs within the immune microenvironment associated with OP. Our findings revealed pronounced expression of various immune cell types in two distinct clusters. Cluster 1 showed significant levels of B cells naive, T cells CD4 memory resting, T cells follicular helper, T cells gamma delta, activated NK cells, M1 Macrophages, resting and activated Dendritic cells, and activated Mast cells. Conversely, Cluster 2 was characterized by abundant B cells memory, Plasma cells, T cells CD8, naive and activated CD4 memory T cells, resting NK cells, Monocytes, M2 Macrophages, resting Mast cells, and Neutrophils. These observations underscore a critical association between PyMGs pathophysiology, inflammation, and the immune response in OP, thereby providing valuable insights into the disease’s underlying mechanisms.

The quest for biomarkers associated with OP has traditionally been constrained within the scientific literature. Nonetheless, the advent of bioinformatic analyses in recent years has cast light on the nexus between metabolism and OP. Mo et al. unearthed six potential biomarkers (*COL1A1, IBSP, CTSP, CTSD, RAC2, MAF,* and *THBS1*) for OP, through the construction of a predictive model [47–49]. Complementing this, Liu et al. pinpointed *PRKCB, GSDMD, ARMCX3,* and *CASP3* as central genes, suggesting their profound implications for OP prognosis and therapeutic intervention. Despite these advances, a discernible lacuna persists in our understanding of PyM involvement in OP, which our research endeavours to bridge. Our study embarks on an examination of cellular metabolic processes to delineate effective therapeutic modalities for OP. Diverging from the trajectory of previous investigations, our methodology adopts an innovative approach by harnessing an expanded set of PyMGs derived from the GEO, thus broadening our analytical horizon. Our model not only provides a theoretical framework but also charts a course for future metabolic research and potential metabolic-targeted therapies in OP. However, our study has its limitations. The underlying mechanisms of OP necessitate further exploration through extensive in vivo and in vitro studies. The prognostic significance of PyMGs within the OP context remains elusive, opening avenues for future research to uncover the roles of PyMGs in OP’s etiology and progression. This area presents a fertile ground for ongoing scholarly engagement and innovation.

## Conclusions

OP is defined by a sophisticated nexus of molecular targets, signaling cascades, and regulatory mechanisms, characterized by their synergistic and reciprocal modulation. Central to this complexity may are PyMGs, key regulators of the synthesis of essential molecules such as *IGKC, TMEM187, RPS11, IGLL3P,* and *GOLGA8N*, which critically influence signaling pathways related to calcium, chemokines, and epsilon receptor interactions.These focused research strategies aim to expand our knowledge of OP and spearhead the creation of innovative therapeutic options.

### Supplementary Information

**Supplementary Material 1.**

## Data Availability

The datasets generated during and/or analyzed during the current study are available in the appendix.

## Abbreviations

OP: Osteoporosis
GO: Gene Ontology
TCM: Traditional Chinese medicine
MF: Molecular functions
KEGG: Kyoto Encyclopedia of Genes and Genomes
GEO: Gene Expression Omnibus
PyMGs: Pyrimidine metabolism genes
BP: Biological processes
CC: Cellular components
DEGs: Differentially Expressed Genes
