# Supplementary material for: Integration of bioinformatics and machine learning approaches for the validation of pyrimidine metabolism-related genes and their implications in immunotherapy for osteoporosis
Source: BMC Musculoskelet Disord. 2024 May 22;25:402. doi: 10.1186/s12891-024-07512-z (PMC11110368; doi:10.1186/s12891-024-07512-z)
Supplement: Supplementary file 1 — Supplementary Material 1. [file 12891_2024_7512_MOESM1_ESM.doc]

Integration of Bioinformatics and Machine Learning Approaches for the Validation of Pyrimidine Metabolism-Related Genes and Their Implications in Immunotherapy for Osteoporosis

**Supplementary appendix to the manuscript**

Contents of supplementary appendix

[Appendix 1 3](#__RefHeading___Toc4150)

[Datasets and Pyrimidine Metabolism genes 3](#__RefHeading___Toc21977)

[Table S1. Pyrimidine Metabolism genes 3](#__RefHeading___Toc19729)

[Appendix 2 4](#__RefHeading___Toc27955)

[DEGs linked to PyMGs 4](#__RefHeading___Toc8414)

[Table S2. 37 DEGs linked to PyMGs. 4](#__RefHeading___Toc6491)

[Appendix 3 6](#__RefHeading___Toc18006)

[Table S3. chromosomal positions of PyMGs. 6](#__RefHeading___Toc18753)

[Appendix 4 10](#__RefHeading___Toc31581)

[InterGenes 10](#__RefHeading___Toc27424)

[Table S4. InterGenes. 10](#__RefHeading___Toc9604)

[Appendix 5 14](#__RefHeading___Toc17792)

[ImportanceGene.XGB 14](#__RefHeading___Toc29572)

[Table S5. ImportanceGene.XGB. 14](#__RefHeading___Toc3393)

[Appendix 6 15](#__RefHeading___Toc11167)

[corResult 15](#__RefHeading___Toc18677)

[Table S6. corResult. 15](#__RefHeading___Toc5649)

[Appendix 7 16](#__RefHeading___Toc21526)

[Table S7. Drug prediction. 16](#__RefHeading___Toc11248)

# Appendix 1

**Datasets and Pyrimidine Metabolism genes**

**Table S1. Pyrimidine Metabolism genes**

| CAD | POLR1D | DCTD | ENPP1 | TWISTNB |
| --- | --- | --- | --- | --- |
| DHODH | POLR3H | TK2 | ENPP3 | ENTPD6 |
| UMPS | POLR3GL | TK1 | TXNRD1 | CTPS1 |
| CMPK1 | POLR3G | DTYMK | TXNRD2 | CTPS2 |
| CMPK2 | POLR3F | NUDT2 | TXNRD3 | POLR1A |
| NME6 | POLA1 | POLR2H | RRM1 | POLR1B |
| NME7 | POLA2 | POLR2I | RRM2B | ZNRD1 |
| NME2 | PRIM1 | POLR2L | RRM2 | POLR2C |
| NME4 | PRIM2 | POLR2J | DCTPP1 | POLR2D |
| NME1 | POLD1 | POLR2J3 | DUT | POLR2E |
| NME3 | POLD2 | POLR2J2 | TYMS | POLR2F |
| NME1-NME2 | POLD3 | POLR2K | CDA | POLR2G |
| AK9 | POLD4 | POLR3A | TYMP | POLR1E |
| PNPT1 | POLE | POLR3B | PNP | UPP1 |
| ENTPD3 | POLE2 | POLR3C | DCK | UPRT |
| ENTPD8 | POLE3 | POLR3D | NT5C2 | DPYD |
| ENTPD1 | POLE4 | POLR3E | NT5C1A | DPYS |
| CANT1 | UCK1 | RPC5 | NT5C1B | UPB1 |
| ENTPD4 | UCK2 | POLR1C | NT5C | POLR2A |
| ENTPD5 | UCKL1 | POLR3K | NT5M | NT5C3A |
| POLR2B | UPP2 | NT5E | NT5C1B-RDH14 | NT5C3B |

# Appendix 2

## **DEGs linked to PyMGs**

**Table S2. 37 DEGs linked to PyMGs.**

| ID | GSM40049 | GSM40052 | GSM40202 | GSM40204 | GSM40208 |
| --- | --- | --- | --- | --- | --- |
| CAD | 5.080698 | 5.163256 | 5.121294 | 5.167224 | 5.248144 |
| UMPS | 5.624325 | 5.308285 | 5.557076 | 5.320559 | 5.415491 |
| NME6 | 3.657594 | 3.732333 | 3.670708 | 3.584654 | 3.621298 |
| NME4 | 2.268645 | 2.27589 | 2.271669 | 2.272345 | 2.27302 |
| ENTPD3 | 7.936785 | 7.767279 | 7.660985 | 7.889876 | 7.613442 |
| ENTPD1 | 2.268645 | 2.27589 | 2.271669 | 2.272345 | 2.27302 |
| CANT1 | 3.821283 | 3.811707 | 3.764318 | 3.918734 | 3.847563 |
| ENTPD4 | 2.268645 | 2.27589 | 2.271669 | 2.272345 | 2.27302 |
| POLR2A | 5.133534 | 5.019256 | 5.183447 | 5.180754 | 5.031422 |
| POLR2C | 2.268645 | 2.27589 | 2.271669 | 2.272345 | 2.27302 |
| POLR2I | 2.268645 | 2.27589 | 2.271669 | 2.272345 | 2.27302 |
| POLR2L | 2.268645 | 2.27589 | 2.271669 | 2.272345 | 2.27302 |
| POLR2K | 2.268645 | 2.27589 | 2.271669 | 2.272345 | 2.27302 |
| POLR3B | 5.272564 | 5.377888 | 5.303809 | 5.197704 | 5.184646 |
| POLR3C | 2.268645 | 2.27589 | 2.271669 | 2.272345 | 2.27302 |
| POLR3K | 3.752503 | 3.678609 | 3.617657 | 3.602658 | 3.738673 |
| POLR1D | 2.268645 | 2.27589 | 2.271669 | 2.272345 | 2.27302 |
| POLA1 | 5.645154 | 5.670084 | 5.701583 | 5.64739 | 5.619189 |
| PRIM1 | 5.722279 | 5.62263 | 5.693119 | 5.701583 | 5.498481 |
| POLD1 | 3.650439 | 3.614821 | 3.565312 | 3.636374 | 3.645172 |
| POLD2 | 3.856113 | 3.842044 | 3.87941 | 3.846862 | 3.787131 |
| POLD3 | 4.648141 | 4.598777 | 4.725406 | 4.59145 | 4.53329 |
| POLE | 6.042288 | 5.899896 | 5.890514 | 5.937144 | 6.012434 |
| NT5C2 | 2.268645 | 2.27589 | 2.271669 | 2.272345 | 2.27302 |
| NT5M | 4.453839 | 4.344964 | 4.391992 | 4.407949 | 4.445214 |
| UPP1 | 2.268645 | 2.27589 | 2.271669 | 2.272345 | 2.27302 |
| TXNRD2 | 4.76312 | 4.790342 | 4.805428 | 4.843991 | 4.946134 |
| RRM1 | 2.268645 | 2.27589 | 2.271669 | 2.272345 | 2.27302 |
| RRM2 | 2.268645 | 2.27589 | 2.271669 | 2.272345 | 2.27302 |
| DCTPP1 | 2.268645 | 2.27589 | 2.271669 | 2.272345 | 2.27302 |
| DUT | 2.268645 | 2.27589 | 2.271669 | 2.272345 | 2.27302 |
| TYMS | 4.694422 | 4.76312 | 4.712419 | 4.836296 | 4.709495 |
| CDA | 2.268645 | 2.27589 | 2.271669 | 2.272345 | 2.27302 |
| TYMP | 2.268645 | 2.27589 | 2.271669 | 2.272345 | 2.27302 |
| DCTD | 2.268645 | 2.27589 | 2.271669 | 2.272345 | 2.27302 |
| TK2 | 3.689792 | 3.69861 | 3.627659 | 3.733843 | 3.699384 |
| NUDT2 | 2.268645 | 2.27589 | 2.271669 | 2.272345 | 2.27302 |

# Appendix 3

**Chromosomal positions of PyMGs**

**Table S3. chromosomal positions of PyMGs.**

| Chromosome | chromStart | chromEnd | Gene |
| --- | --- | --- | --- |
| chr1 | 20588948 | 20618908 | CDA |
| chr1 | 39659121 | 39672038 | NT5C1A |
| chr1 | 40979335 | 41012565 | CTPS1 |
| chr1 | 47333797 | 47378839 | CMPK1 |
| chr1 | 97077743 | 97921049 | DPYD |
| chr1 | 145824088 | 145842505 | POLR3C |
| chr1 | 145964702 | 145978848 | POLR3GL |
| chr1 | 165827531 | 165911618 | UCK2 |
| chr1 | 169132531 | 169367967 | NME7 |
| chr2 | 6840570 | 6866635 | CMPK2 |
| chr2 | 10122328 | 10131419 | RRM2 |
| chr2 | 18555545 | 18589564 | NT5C1B-RDH14 |
| chr2 | 18562872 | 18589572 | NT5C1B |
| chr2 | 27217390 | 27243943 | CAD |
| chr2 | 55634265 | 55693910 | PNPT1 |
| chr2 | 74958492 | 74970128 | POLE4 |
| chr2 | 86020216 | 86106155 | POLR1A |
| chr2 | 112541915 | 112577150 | POLR1B |
| chr2 | 127843551 | 127858157 | POLR2D |
| chr2 | 157876702 | 158136154 | UPP2 |
| chr2 | 241675742 | 241686991 | DTYMK |
| chr3 | 40387156 | 40428619 | ENTPD3 |
| chr3 | 48293264 | 48301685 | NME6 |
| chr3 | 124730366 | 124749273 | UMPS |
| chr3 | 126571779 | 126655155 | TXNRD3 |
| chr3 | 184361718 | 184368596 | POLR2H |
| chr4 | 56977722 | 57031168 | POLR2B |
| chr4 | 70992538 | 71030914 | DCK |
| chr4 | 182890060 | 182917936 | DCTD |
| chr5 | 90471748 | 90514553 | POLR3G |
| chr6 | 30058899 | 30064909 | ZNRD1 |
| chr6 | 43509702 | 43529585 | POLR1C |
| chr6 | 57314805 | 57646849 | PRIM2 |
| chr6 | 85449584 | 85495791 | NT5E |
| chr6 | 109492856 | 109691217 | AK9 |
| chr6 | 131628442 | 131747418 | ENPP3 |
| chr6 | 131808016 | 131895155 | ENPP1 |
| chr7 | 19695462 | 19709087 | TWISTNB |
| chr7 | 33014114 | 33062797 | NT5C3A |
| chr7 | 44114681 | 44124358 | POLD2 |
| chr7 | 48088628 | 48108733 | UPP1 |
| chr7 | 102473118 | 102478907 | POLR2J |
| chr7 | 102537918 | 102572656 | POLR2J3 |
| chr7 | 102665368 | 102671629 | POLR2J2 |
| chr8 | 22245104 | 22254600 | POLR3D |
| chr8 | 23385783 | 23457695 | ENTPD4 |
| chr8 | 100150584 | 100154002 | POLR2K |
| chr8 | 102204502 | 102239118 | RRM2B |
| chr8 | 104330324 | 104467053 | DPYS |
| chr9 | 34329506 | 34343713 | NUDT2 |
| chr9 | 37485935 | 37503697 | POLR1E |
| chr9 | 113407235 | 113410672 | POLE3 |
| chr9 | 131523801 | 131531268 | UCK1 |
| chr9 | 137434364 | 137441816 | ENTPD8 |
| chr10 | 77969251 | 78029545 | POLR3A |
| chr10 | 95711779 | 95869695 | ENTPD1 |
| chr10 | 103088017 | 103193306 | NT5C2 |
| chr11 | 837356 | 842545 | POLR2L |
| chr11 | 4094707 | 4138876 | RRM1 |
| chr11 | 62761544 | 62766710 | POLR2G |
| chr11 | 65261762 | 65305589 | POLA2 |
| chr11 | 67350777 | 67356972 | POLD4 |
| chr11 | 74493851 | 74669117 | POLD3 |
| chr12 | 56731596 | 56752373 | PRIM1 |
| chr12 | 104215779 | 104350305 | TXNRD1 |
| chr12 | 106357658 | 106510198 | POLR3B |
| chr12 | 132623753 | 132687365 | POLE |
| chr13 | 27620742 | 27667415 | POLR1D |
| chr14 | 20468954 | 20477094 | PNP |
| chr14 | 49643555 | 49688422 | POLE2 |
| chr14 | 73958010 | 74019399 | ENTPD5 |
| chr15 | 48331011 | 48343373 | DUT |
| chr16 | 46407 | 53628 | POLR3K |
| chr16 | 396725 | 410367 | NME4 |
| chr16 | 1770286 | 1771730 | NME3 |
| chr16 | 22297375 | 22335103 | POLR3E |
| chr16 | 30423619 | 30430075 | DCTPP1 |
| chr16 | 57462387 | 57472010 | POLR2C |
| chr16 | 66508003 | 66552544 | TK2 |
| chr16 | 72008588 | 72027664 | DHODH |
| chr17 | 7484366 | 7514618 | POLR2A |
| chr17 | 17303335 | 17347663 | NT5M |
| chr17 | 41825181 | 41836263 | NT5C3B |
| chr17 | 51153536 | 51162428 | NME1 |
| chr17 | 51153590 | 51171744 | NME1-NME2 |
| chr17 | 51165435 | 51171747 | NME2 |
| chr17 | 75130225 | 75131795 | NT5C |
| chr17 | 78174075 | 78187233 | TK1 |
| chr17 | 78991717 | 79009867 | CANT1 |
| chr18 | 657604 | 673578 | TYMS |
| chr19 | 1086579 | 1095380 | POLR2E |
| chr19 | 36113710 | 36115346 | POLR2I |
| chr19 | 50384204 | 50418018 | POLD1 |
| chr20 | 18467127 | 18484643 | POLR3F |
| chr20 | 25195693 | 25226729 | ENTPD6 |
| chr20 | 63939829 | 63956415 | UCKL1 |
| chr22 | 19875517 | 19941992 | TXNRD2 |
| chr22 | 24494107 | 24528390 | UPB1 |
| chr22 | 37952607 | 38041915 | POLR2F |
| chr22 | 41525804 | 41544606 | POLR3H |
| chr22 | 50525752 | 50530056 | TYMP |
| chrX | 16588003 | 16712936 | CTPS2 |
| chrX | 24693919 | 24996986 | POLA1 |
| chrX | 75274085 | 75304600 | UPRT |

# Appendix 4

**InterGenes**

**Table S4. InterGenes.**

| DAPK1 | FHL1 | DUT | CUTA | SUB1 | MYL6 |
| --- | --- | --- | --- | --- | --- |
| UNC50 | ABCF3 | IGLL3P | RNF44 | RAC1 | Y16709 |
| WASF2 | STAU1 | TINF2 | TNFAIP8 | CCR2 | HLA-DPB1 |
| MLF2 | RASSF4 | DDX39A | PUM1 | FYN | HUWE1 |
| TMEM187 | LSM7 | ATOX1 | LILRA2 | MRPL33 | RPL18 |
| IQGAP2 | CLINT1 | GLTP | RBL2 | SLC38A2 | TUBA1B |
| FAM134B | CITED2 | XBP1 | GNB2 | LBR | PLBD1 |
| TSPYL2 | YY1AP1 | HADHA | UBE4A | STARD7 | ANXA2 |
| BTG3 | FDFT1 | BASP1 | CCT8 | TPI1 | IFITM2 |
| PUF60 | PAQR6 | YY1 | PSMB4 | TPST2 | TUBA1C |
| MBD4 | OGT | IL6R | TMED2 | NONO | HLA-DPA1 |
| ABCG4 | KLK1 | CRIP1 | WRB | NDUFA1 | S100A11 |
| SMS | THBS3 | NCKAP1L | RBM3 | EEF1D | RPS27A |
| OSTF1 | CETN1 | RPN1 | STAT6 | HMGB2 | KCTD12 |
| EPHA2 | RNF4 | ESD | TRAPPC8 | ARRB2 | RPS21 |
| TMPRSS11D | TLR1 | FAM129A | PNN | ICAM3 | RPL31 |
| NPY | CTRC | NDUFA13 | EIF4B | SF3B1 | SH3BGRL3 |
| SPHK1 | INTS8 | TES | TIMP2 | LGALS2 | MYL12B |
| CCDC28A | VWF | SNRPB | ANP32A | MYH9 | LGALS1 |
| TEX30 | PSMC5 | MDH2 | PSMA2 | CD93 | PFN1 |
| SLC6A4 | BTBD1 | SRSF3 | HNRNPC | GSTP1 | RPL37A |
| RP11-10N23.4 | EIF2B1 | SRPR | CAST | PGK1 | RPL12 |
| HTR6 | IL1RAPL2 | CHP1 | RALB | RTN3 | HLA-G |
| S100A2 | GANAB | SH3BP5 | ATP5G3 | CFP | ARPC2 |
| CACNA2D3 | C1QBP | TAF7 | ATP5G2 | PPA1 | NACA |
| IGKC | MBD2 | SERINC5 | CAPG | EIF4H | S100A12 |
| CDC37 | ACAA1 | LAT2 | PCBP2 | ATP5J | EEF2 |
| TCF25 | CNOT8 | MGAT1 | EFHD2 | CSF3R | RPLP0 |
| SLC9A3R1 | ENOPH1 | FCGR3B | CANX | H2AFY | RPL24 |
| GCHFR | RNF146 | RAB1B | CDIPT | SORL1 | TXNIP |
| SPSB3 | PPM1B | NCL | HK3 | HLA-DMA | RPS25 |
| CNPY3 | RARRES3 | LMO2 | RXRA | PPIB | CTSS |
| TBCB | NDUFV2 | GRB2 | GNAI2 | CD1D | RPL6 |
| STX12 | MICB | LILRA6 | CERS2 | CAPN2 | RPS10 |
| IST1 | RNPS1 | CDV3 | RAP1A | CYBA | S100A8 |
| LRMP | SEPW1 | MAN2B1 | SRSF7 | KARS | HLA-C |
| FAM134C | RPS6KA3 | SYK | SELPLG | IFNGR1 | LYZ |
| PIN1 | CCDC71 | UBE2NL | GYG1 | CAPZB | PPBP |
| ZNF106 | MEA1 | FRAT2 | PCMT1 | APLP2 | CDK5 |
| KRT10 | IGF2R | CBX4 | BST1 | TLR8 | LITAF |
| CASP8 | SNRPD3 | SF3B4 | HNRNPR | ANXA2P2 | SCAF8 |
| TULP2 | PRPF4B | EGR2 | HDAC1 | SSR4 | RPS6KA1 |
| EXTL3 | SDF4 | DUSP22 | GIMAP6 | SEC22B | CRBN |
| IL3RA | MARCO | QPCT | SUMO4 | H2AFZ | TBC1D9 |
| PLK3 | RTN1 | ARL6IP1 | HSP90AB1 | ALOX5AP | CLTA |
| NR1H3 | GLUD1 | HNRNPD | CXCR4 | SNRPD2 | ARCN1 |
| RAB6B | VPS51 | BANF1 | ATP5I | RPL36AL | PRKCD |
| GS1-111G14.1 | POLR2L | UBE2N | PKM | IFITM1 | KHDRBS1 |
| CLUH | CD4 | NOTCH2 | CDK5RAP3 | RAB8A | CTDSP1 |
| RAB3D | M6PR | FLI1 | SON | RPL35A | ARF3 |
| SEC23B | CXCR2 | MYCBP2 | ABHD17A | IQGAP1 | TMEM230 |
| CTNNA1 | SNRNP27 | DEGS1 | CDC42 | CST3 | IL17RA |
| CIDEB | RPA2 | LSM1 | COX5B | RNASE2 | VAMP3 |
| IRF2BP1 | MAP3K5 | CTDSP2 | FBL | PTPN6 | PECAM1 |
| AIP | RPS11 | HP1BP3 | DPM1 | GIMAP4 | RPS14 |
| ARG2 | PRPF40A | ATP6V1E1 | RAB27A | TPP1 | RPS9 |
| KDELR2 | BAG6 | SEC61G | LSP1 | XIST | CD52 |
| LYZL6 | HBEGF | CNN2 | HMGN1 | C14orf2 | RPL38 |
| PES1 | HSD17B10 | ARF6 | HMGN4 | COX4I1 | LUC7L3 |
| SERPINA4 | HBD | NADK | FCER1A | PTPRO | EIF6 |
| STRA6 | UBA1 | PDIA6 | GOLGA8N | CSF1R | POLR1D |
| S100A1 | CSRP1 | DOK3 | DHX15 | NPM1 | GNB5 |
| HNRNPA3 | RNF114 | RAN | DAD1 | RNASE6 | LILRA1 |
| ETHE1 | KDELR1 | PRKCSH | SEPT9 | ADD3 | NDUFB8 |
| IRAK1 | PHLDA1 | MAPKAPK3 | ARHGEF6 | RHOG | HPCAL1 |
| CARTPT | LSM14A | NAGA | TOP2B | COX7C | IL2RG |
| FAM189B | SRRM1 | EIF4A3 | DDX17 | FLNA | HHEX |
| PARP8 | PSMA7 | TGOLN2 | PRR11 | CLC | CD33 |
| IL1A | SAP18 | ARID1A | P4HB | CD164 | IRF9 |
| CD47 | KDM3B | HNRNPDL | GZMB | 15-Sep | EDF1 |
| LOC102723620 | FLII | ACADVL | EID1 | CX3CR1 | VASP |
| PARD3 | SFT2D2 | CTR9 | CD163 | CCNI | CD37 |
| NOD2 | PDHB | TMED10 | WIPF1 | MORF4L1 | GOLPH3 |
| GPR21 | UBE2L3 | CNIH1 | RNASET2 | IGLC1 | P2RY13 |
| PSMC6 | EPS15 | CD55 | FKBP5 | DPYSL2 | CSF2RB |
| UBE2G1 | SNW1 | CRTAP | ATP6V0E1 | FXYD5 | MYO1F |
| WDR11 | PSMD4 | OAT | HINT1 | PLP2 | RAC2 |
| DNAJC1 | NDUFA3 | GPR65 | SLA | IGJ | DUSP6 |
| SLC15A3 | PTAFR | DYSF | GLIPR1 | PPP1CC | SIRT1 |
| MPP2 | MYL12A | SIDT2 | ZNF217 | LST1 | TMEM9B |
| ZBTB18 | METTL7A | DENND4B | RAD21 | ATP5L | KPNB1 |
| LEFTY1 | RAB8B | METTL9 | CYTIP | YWHAB | PRPF8 |
| AIDA | PIK3CD | TMED9 | LAMP1 | RPS28 | UQCRQ |
| CD81 | HLA-DQB1 | YWHAE | YWHAQ | ITGAM | CALHM2 |
| CECR5 | FNTA | SRSF11 | CSK | GDI2 | ITGAL |
| SS18L2 | CAB39 | AHNAK | EIF2S3 | JTB | SASH3 |
| IK | UBL5 | DCTN3 | ENO1 | RPL14 | UCP2 |
| B4GALT2 | HMHA1 | MAPRE1 | PRNP | RPL15 | RPL36 |
| BRD2 | NPIPA1 | SERBP1 | GNS | HLA-F | HLA-J |
| PLXNC1 | CEBPA | TOB1 | DBI |  |  |

# Appendix 5

**ImportanceGene.XGB**

**Table S5. ImportanceGene.XGB.**

| variable | permutation | dropout_loss | label |
| --- | --- | --- | --- |
| IGKC | 0 | 0.329836334 | XGB |
| TMEM187 | 0 | 0.33142518 | XGB |
| RPS11 | 0 | 0.332909296 | XGB |
| IGLL3P | 0 | 0.333258659 | XGB |
| GOLGA8N | 0 | 0.383312463 | XGB |

# Appendix 6

**corResult**

**Table S6. corResult.**

| Gene | Clinical | cor | pvalue |
| --- | --- | --- | --- |
| IGKC | Age | 0.6 | 0.35 |
| TMEM187 | Age | -0.2 | 0.783333333 |
| RPS11 | Age | -0.5 | 0.45 |
| IGLL3P | Age | 0.6 | 0.35 |
| GOLGA8N | Age | -0.3 | 0.683333333 |

# Appendix 7

**Drug prediction**

**Table S7. Drug prediction.**

| search_term | gene | drug | interaction_types | sources |
| --- | --- | --- | --- | --- |
| CSF3R | CSF3R | FILGRASTIM | stimulator|agonist | TdgClinicalTrial|ChemblInteractions|TEND|TTD |
| CXCR4 | CXCR4 | PLERIXAFOR | partial agonist|antagonist | DTC|TdgClinicalTrial|ChemblInteractions|NCI|TEND|TTD |
| SLC6A4 | SLC6A4 | SERTRALINE | negative modulator|binder|inhibitor | TdgClinicalTrial|TEND |
| CACNA2D3 | CACNA2D3 | PREGABALIN | modulator | ChemblInteractions |
| CACNA2D3 | CACNA2D3 | GABAPENTIN | modulator | ChemblInteractions |
| CACNA2D3 | CACNA2D3 | GABAPENTIN ENACARBIL | modulator | ChemblInteractions |
| NOD2 | NOD2 | MIFAMURTIDE | ligand | ChemblInteractions |
| FYN | FYN | DASATINIB | inhibitor|multitarget | TdgClinicalTrial|ChemblInteractions|TEND|TTD |
| EPHA2 | EPHA2 | DASATINIB | inhibitor|antagonist | DTC|TdgClinicalTrial|ChemblInteractions|TEND|DoCM|MyCancerGenomeClinicalTrial |
| EPHA2 | EPHA2 | VANDETANIB | inhibitor | ChemblInteractions |
| EPHA2 | EPHA2 | REGORAFENIB | inhibitor | MyCancerGenomeClinicalTrial |
| SLC6A4 | SLC6A4 | LEVOMILNACIPRAN | inhibitor | TdgClinicalTrial |
| SLC6A4 | SLC6A4 | AMOXAPINE | inhibitor | TdgClinicalTrial|TEND |
| SLC6A4 | SLC6A4 | DAPOXETINE | inhibitor | TdgClinicalTrial |
| SLC6A4 | SLC6A4 | CLOMIPRAMINE | inhibitor | TdgClinicalTrial|TEND|PharmGKB |
| SLC6A4 | SLC6A4 | DESVENLAFAXINE | inhibitor | TdgClinicalTrial|TEND |
| SLC6A4 | SLC6A4 | NEFAZODONE | inhibitor | TdgClinicalTrial|TEND |
| SLC6A4 | SLC6A4 | AMITRIPTYLINE | inhibitor | TdgClinicalTrial|TEND |
| SLC6A4 | SLC6A4 | FLUOXETINE | inhibitor | TdgClinicalTrial|NCI|TEND |
| SLC6A4 | SLC6A4 | DULOXETINE | inhibitor | TdgClinicalTrial|TEND |
| SLC6A4 | SLC6A4 | DOXEPIN | inhibitor | TdgClinicalTrial|TEND |
| SLC6A4 | SLC6A4 | TRAZODONE | inhibitor | TdgClinicalTrial|TEND |
| SLC6A4 | SLC6A4 | CITALOPRAM | inhibitor | TdgClinicalTrial|TEND |
| SLC6A4 | SLC6A4 | METHYLPHENIDATE | inhibitor | TdgClinicalTrial|TEND |
| SLC6A4 | SLC6A4 | MILNACIPRAN | inhibitor | TdgClinicalTrial|TEND |
| SLC6A4 | SLC6A4 | ESCITALOPRAM | inhibitor | TdgClinicalTrial|TEND |
| SLC6A4 | SLC6A4 | PROTRIPTYLINE | inhibitor | TdgClinicalTrial|TEND |
| SLC6A4 | SLC6A4 | TRIMIPRAMINE | inhibitor | TdgClinicalTrial|TEND |
| SLC6A4 | SLC6A4 | PSEUDOEPHEDRINE | inhibitor | TdgClinicalTrial |
| SLC6A4 | SLC6A4 | VORTIOXETINE | inhibitor | TdgClinicalTrial |
| SLC6A4 | SLC6A4 | IMIPRAMINE | inhibitor | TdgClinicalTrial|TEND |
| SLC6A4 | SLC6A4 | PAROXETINE | inhibitor | TdgClinicalTrial|TEND |
| SLC6A4 | SLC6A4 | FLUVOXAMINE | inhibitor | TdgClinicalTrial|TEND |
| SLC6A4 | SLC6A4 | VENLAFAXINE | inhibitor | TdgClinicalTrial|TEND |
| SLC6A4 | SLC6A4 | NORTRIPTYLINE | inhibitor | TdgClinicalTrial|TEND |
| SLC6A4 | SLC6A4 | DESIPRAMINE | inhibitor | TdgClinicalTrial|TEND |
| SLC6A4 | SLC6A4 | COCAINE | inhibitor | TdgClinicalTrial|TEND |
| SLC6A4 | SLC6A4 | PHENTERMINE | inhibitor | TdgClinicalTrial|TEND |
| SLC6A4 | SLC6A4 | TRAMADOL | inhibitor | TdgClinicalTrial|TEND |
| PSMC6 | PSMC6 | CARFILZOMIB | inhibitor | DTC|MyCancerGenome|ChemblInteractions |
| PSMC6 | PSMC6 | IXAZOMIB CITRATE | inhibitor | ChemblInteractions |
| PSMC6 | PSMC6 | BORTEZOMIB | inhibitor | DTC|MyCancerGenome|ChemblInteractions |
| VWF | VWF | CAPLACIZUMAB | inhibitor | TdgClinicalTrial|ChemblInteractions|TTD |
| PSMC5 | PSMC5 | CARFILZOMIB | inhibitor | DTC|MyCancerGenome|ChemblInteractions |
| PSMC5 | PSMC5 | BORTEZOMIB | inhibitor | DTC|MyCancerGenome|ChemblInteractions |
| PSMC5 | PSMC5 | IXAZOMIB | inhibitor | MyCancerGenome |
| PSMC5 | PSMC5 | IXAZOMIB CITRATE | inhibitor | ChemblInteractions |
| NDUFV2 | NDUFV2 | METFORMIN HYDROCHLORIDE | inhibitor | ChemblInteractions |
| PSMA7 | PSMA7 | CARFILZOMIB | inhibitor | DTC|MyCancerGenome|ChemblInteractions |
| PSMA7 | PSMA7 | IXAZOMIB CITRATE | inhibitor | ChemblInteractions |
| PSMA7 | PSMA7 | BORTEZOMIB | inhibitor | DTC|MyCancerGenome|ChemblInteractions |
| PSMD4 | PSMD4 | CARFILZOMIB | inhibitor | DTC|MyCancerGenome|ChemblInteractions|CIViC |
| PSMD4 | PSMD4 | IXAZOMIB CITRATE | inhibitor | ChemblInteractions |
| PSMD4 | PSMD4 | BORTEZOMIB | inhibitor | DTC|MyCancerGenome|ChemblInteractions |
| NDUFA3 | NDUFA3 | METFORMIN HYDROCHLORIDE | inhibitor | ChemblInteractions |
| PIK3CD | PIK3CD | COPANLISIB | inhibitor | MyCancerGenome|ChemblInteractions|MyCancerGenomeClinicalTrial |
| PIK3CD | PIK3CD | DUVELISIB | inhibitor | ChemblInteractions|TTD |
| PIK3CD | PIK3CD | ALPELISIB | inhibitor | MyCancerGenome|MyCancerGenomeClinicalTrial |
| PIK3CD | PIK3CD | IDELALISIB | inhibitor | MyCancerGenome|TdgClinicalTrial|ChemblInteractions|TTD |
| NDUFB8 | NDUFB8 | METFORMIN HYDROCHLORIDE | inhibitor | ChemblInteractions |
| IL2RG | IL2RG | DACLIZUMAB | inhibitor | ChemblInteractions |
| IL2RG | IL2RG | BASILIXIMAB | inhibitor | ChemblInteractions |
| CRBN | CRBN | POMALIDOMIDE | inhibitor | ChemblInteractions|CIViC |
| CRBN | CRBN | LENALIDOMIDE | inhibitor | ChemblInteractions|CIViC |
| CRBN | CRBN | THALIDOMIDE | inhibitor | ChemblInteractions |
| NDUFA13 | NDUFA13 | METFORMIN HYDROCHLORIDE | inhibitor | ChemblInteractions |
| SYK | SYK | FOSTAMATINIB | inhibitor | MyCancerGenome|TdgClinicalTrial|ChemblInteractions|TTD |
| PRKCD | PRKCD | MIDOSTAURIN | inhibitor | ChemblInteractions |
| PSMB4 | PSMB4 | CARFILZOMIB | inhibitor | DTC|MyCancerGenome|ChemblInteractions |
| PSMB4 | PSMB4 | BORTEZOMIB | inhibitor | DTC|MyCancerGenome|ChemblInteractions |
| PSMB4 | PSMB4 | IXAZOMIB CITRATE | inhibitor | ChemblInteractions |
| PSMA2 | PSMA2 | BORTEZOMIB | inhibitor | DTC|MyCancerGenome|ChemblInteractions |
| PSMA2 | PSMA2 | CARFILZOMIB | inhibitor | DTC|MyCancerGenome|ChemblInteractions |
| PSMA2 | PSMA2 | IXAZOMIB CITRATE | inhibitor | ChemblInteractions |
| HDAC1 | HDAC1 | PANOBINOSTAT | inhibitor | TALC|DTC|MyCancerGenome|TdgClinicalTrial|CancerCommons|TTD |
| HDAC1 | HDAC1 | VORINOSTAT | inhibitor | TALC|DTC|MyCancerGenome|TdgClinicalTrial|ChemblInteractions|TEND|TTD |
| HDAC1 | HDAC1 | BELINOSTAT | inhibitor | TALC|DTC|MyCancerGenome|TdgClinicalTrial|ChemblInteractions |
| HDAC1 | HDAC1 | PANOBINOSTAT LACTATE | inhibitor | ChemblInteractions |
| FCER1A | FCER1A | OMALIZUMAB | inhibitor | TdgClinicalTrial |
| TOP2B | TOP2B | ETOPOSIDE | inhibitor | DTC|ChemblInteractions|TTD |
| TOP2B | TOP2B | TENIPOSIDE | inhibitor | ChemblInteractions|TTD |
| TOP2B | TOP2B | DAUNORUBICIN | inhibitor | TdgClinicalTrial |
| P4HB | P4HB | LOMITAPIDE MESYLATE | inhibitor | ChemblInteractions |
| FYN | FYN | NINTEDANIB | inhibitor | TALC |
| NDUFA1 | NDUFA1 | METFORMIN HYDROCHLORIDE | inhibitor | ChemblInteractions |
| CSF1R | CSF1R | DASATINIB | inhibitor | TALC |
| CSF1R | CSF1R | PEXIDARTINIB | inhibitor | MyCancerGenome|JAX-CKB|ChemblInteractions|CIViC|CancerCommons |
| CSF1R | CSF1R | SUNITINIB MALATE | inhibitor | ChemblInteractions |
| CSF1R | CSF1R | PAZOPANIB | inhibitor | DTC |
| CSF1R | CSF1R | SUNITINIB | inhibitor | TEND |
| CSF1R | CSF1R | PAZOPANIB HYDROCHLORIDE | inhibitor | ChemblInteractions |
| TUBA1B | TUBA1B | VINCRISTINE SULFATE | inhibitor | ChemblInteractions |
| TUBA1B | TUBA1B | CABAZITAXEL | inhibitor | ChemblInteractions |
| TUBA1B | TUBA1B | IXABEPILONE | inhibitor | ChemblInteractions |
| TUBA1B | TUBA1B | PACLITAXEL | inhibitor | DTC|ChemblInteractions |
| TUBA1B | TUBA1B | ERIBULIN MESYLATE | inhibitor | ChemblInteractions |
| TUBA1B | TUBA1B | BRENTUXIMAB VEDOTIN | inhibitor | ChemblInteractions |
| TUBA1B | TUBA1B | VINBLASTINE SULFATE | inhibitor | ChemblInteractions |
| TUBA1B | TUBA1B | COLCHICINE | inhibitor | DTC|ChemblInteractions |
| TUBA1B | TUBA1B | TRASTUZUMAB EMTANSINE | inhibitor | ChemblInteractions |
| TUBA1B | TUBA1B | VINORELBINE TARTRATE | inhibitor | ChemblInteractions |
| TUBA1B | TUBA1B | DOCETAXEL | inhibitor | ChemblInteractions |
| TUBA1B | TUBA1B | VINFLUNINE | inhibitor | ChemblInteractions |
| TUBA1C | TUBA1C | BRENTUXIMAB VEDOTIN | inhibitor | ChemblInteractions |
| TUBA1C | TUBA1C | CABAZITAXEL | inhibitor | ChemblInteractions |
| TUBA1C | TUBA1C | COLCHICINE | inhibitor | DTC|ChemblInteractions |
| TUBA1C | TUBA1C | VINCRISTINE SULFATE | inhibitor | ChemblInteractions |
| TUBA1C | TUBA1C | TRASTUZUMAB EMTANSINE | inhibitor | ChemblInteractions |
| TUBA1C | TUBA1C | DOCETAXEL | inhibitor | ChemblInteractions |
| TUBA1C | TUBA1C | VINBLASTINE SULFATE | inhibitor | ChemblInteractions |
| TUBA1C | TUBA1C | VINORELBINE TARTRATE | inhibitor | ChemblInteractions |
| TUBA1C | TUBA1C | ERIBULIN MESYLATE | inhibitor | ChemblInteractions |
| TUBA1C | TUBA1C | IXABEPILONE | inhibitor | ChemblInteractions |
| TUBA1C | TUBA1C | PACLITAXEL | inhibitor | DTC|ChemblInteractions |
| TUBA1C | TUBA1C | VINFLUNINE | inhibitor | ChemblInteractions |
| EEF2 | EEF2 | DENILEUKIN DIFTITOX | inhibitor | ChemblInteractions |
| CACNA2D3 | CACNA2D3 | BEPRIDIL HYDROCHLORIDE | blocker | ChemblInteractions |
| IFNGR1 | IFNGR1 | INTERFERON GAMMA-1B | binder|agonist | TdgClinicalTrial|ChemblInteractions |
| IL3RA | IL3RA | TAGRAXOFUSP | binder | TTD |
| IL1A | IL1A | RILONACEPT | binder | TdgClinicalTrial|TEND |
| IL6R | IL6R | TOCILIZUMAB | antibody|inhibitor | MyCancerGenome|TdgClinicalTrial|ChemblInteractions|TEND|PharmGKB|TTD |
| CD52 | CD52 | ALEMTUZUMAB | antibody|inhibitor | MyCancerGenome|TdgClinicalTrial|ChemblInteractions|NCI|TEND|TTD |
| CD4 | CD4 | IBALIZUMAB | antagonist|inhibitor|antibody | ChemblInteractions|TTD |
| HDAC1 | HDAC1 | ROMIDEPSIN | antagonist|inhibitor | TALC|MyCancerGenome|TdgClinicalTrial|ChemblInteractions|TEND|TTD |
| IL6R | IL6R | SARILUMAB | antagonist|antibody | ChemblInteractions|TTD |
| IL17RA | IL17RA | BRODALUMAB | antagonist|antibody | ChemblInteractions|TTD |
| HTR6 | HTR6 | RISPERIDONE | antagonist | PharmGKB |
| HTR6 | HTR6 | ILOPERIDONE | antagonist | TdgClinicalTrial |
| HTR6 | HTR6 | ASENAPINE | antagonist | TdgClinicalTrial |
| HTR6 | HTR6 | OLANZAPINE | antagonist | TdgClinicalTrial |
| RXRA | RXRA | SULINDAC | antagonist | DTC |
| ITGAL | ITGAL | LIFITEGRAST | antagonist | ChemblInteractions|TTD |
| CSF1R | CSF1R | IMATINIB | antagonist | DTC|TdgClinicalTrial|JAX-CKB|TEND|DoCM|CIViC |
| CARTPT | CARTPT | AMPHETAMINE | agonist | TdgClinicalTrial |
| IL2RG | IL2RG | ALDESLEUKIN | agonist | TdgClinicalTrial|ChemblInteractions|TEND |
| RXRA | RXRA | ACITRETIN | agonist | ChemblInteractions |
| RXRA | RXRA | ALITRETINOIN | agonist | DTC|ChemblInteractions|TEND|TTD |
| RXRA | RXRA | ADAPALENE | agonist | TdgClinicalTrial|TEND |
| CSF3R | CSF3R | LIPEGFILGRASTIM | agonist | ChemblInteractions |
| CSF3R | CSF3R | PEGFILGRASTIM | agonist | TdgClinicalTrial|ChemblInteractions|TEND|TTD |
| CSF2RB | CSF2RB | SARGRAMOSTIM | agonist | TdgClinicalTrial|ChemblInteractions|TEND |
| RXRA | RXRA | BEXAROTENE | activator|agonist | TALC|DTC|TdgClinicalTrial|ChemblInteractions|TEND|TTD |
| DAPK1 | DAPK1 | GEMCITABINE | unknown | PharmGKB |
| EPHA2 | EPHA2 | SORAFENIB | unknown | DTC |
| NPY | NPY | BROMOCRIPTINE | unknown | NCI |
| NPY | NPY | HALOPERIDOL | unknown | NCI |
| SLC6A4 | SLC6A4 | RISPERIDONE | unknown | PharmGKB |
| SLC6A4 | SLC6A4 | QUETIAPINE | unknown | PharmGKB |
| SLC6A4 | SLC6A4 | MORPHINE | unknown | PharmGKB |
| SLC6A4 | SLC6A4 | BUPROPION | unknown | PharmGKB |
| SLC6A4 | SLC6A4 | CLOZAPINE | unknown | PharmGKB |
| SLC6A4 | SLC6A4 | ONDANSETRON | unknown | PharmGKB |
| SLC6A4 | SLC6A4 | RIBAVIRIN | unknown | PharmGKB |
| SLC6A4 | SLC6A4 | HALOPERIDOL | unknown | PharmGKB |
| SLC6A4 | SLC6A4 | SOLRIAMFETOL | unknown | TdgClinicalTrial |
| SLC6A4 | SLC6A4 | BUPRENORPHINE | unknown | PharmGKB |
| SLC6A4 | SLC6A4 | OLANZAPINE | unknown | PharmGKB |
| SLC6A4 | SLC6A4 | METHADONE | unknown | PharmGKB |
| SLC6A4 | SLC6A4 | ALCOHOL | unknown | PharmGKB |
| PIN1 | PIN1 | IRINOTECAN | unknown | PharmGKB |
| PIN1 | PIN1 | OXALIPLATIN | unknown | PharmGKB |
| IL3RA | IL3RA | EPOETIN ALFA | unknown | NCI |
| NR1H3 | NR1H3 | ATENOLOL | unknown | PharmGKB |
| NR1H3 | NR1H3 | BEXAROTENE | unknown | DTC |
| NR1H3 | NR1H3 | VERAPAMIL | unknown | PharmGKB |
| IRAK1 | IRAK1 | SORAFENIB | unknown | DTC |
| IRAK1 | IRAK1 | GEFITINIB | unknown | DTC |
| IRAK1 | IRAK1 | IMATINIB | unknown | DTC |
| CARTPT | CARTPT | INSULIN | unknown | NCI |
| CARTPT | CARTPT | DEXAMETHASONE | unknown | NCI |
| CARTPT | CARTPT | PROGESTERONE | unknown | NCI |
| IL1A | IL1A | OLANZAPINE | unknown | PharmGKB |
| IL1A | IL1A | HYDROXYCHLOROQUINE | unknown | NCI |
| NOD2 | NOD2 | TACROLIMUS | unknown | PharmGKB |
| B4GALT2 | B4GALT2 | ASPIRIN | unknown | PharmGKB |
| B4GALT2 | B4GALT2 | CLOPIDOGREL | unknown | PharmGKB |
| BRD2 | BRD2 | ACETAMINOPHEN | unknown | DTC |
| BRD2 | BRD2 | ALPRAZOLAM | unknown | DTC |
| BRD2 | BRD2 | MIDAZOLAM | unknown | DTC |
| SIRT1 | SIRT1 | NIACINAMIDE | unknown | DTC |
| CDK5 | CDK5 | PALBOCICLIB | unknown | DTC |
| FDFT1 | FDFT1 | LOVASTATIN | unknown | NCI |
| KLK1 | KLK1 | ECALLANTIDE | unknown | TdgClinicalTrial|TEND |
| VWF | VWF | RIBAVIRIN | unknown | NCI |
| VWF | VWF | ACETYLCYSTEINE | unknown | NCI |
| VWF | VWF | MITOMYCIN | unknown | NCI |
| VWF | VWF | PREDNISONE | unknown | NCI |
| VWF | VWF | STREPTOZOCIN | unknown | NCI |
| VWF | VWF | THALIDOMIDE | unknown | NCI |
| VWF | VWF | WARFARIN | unknown | NCI |
| VWF | VWF | PENTOXIFYLLINE | unknown | NCI |
| VWF | VWF | PHENYLEPHRINE | unknown | NCI |
| VWF | VWF | VINCRISTINE | unknown | NCI |
| GANAB | GANAB | MIGLUSTAT | unknown | DTC |
| MICB | MICB | RIBAVIRIN | unknown | PharmGKB |
| RPS6KA3 | RPS6KA3 | UREA | unknown | NCI |
| RPS6KA3 | RPS6KA3 | PALBOCICLIB | unknown | DTC |
| SDF4 | SDF4 | ALCOHOL | unknown | NCI |
| CXCR2 | CXCR2 | CLOTRIMAZOLE | unknown | TTD |
| CXCR2 | CXCR2 | ACETYLCYSTEINE | unknown | NCI |
| CXCR2 | CXCR2 | IBUPROFEN | unknown | TTD |
| MAP3K5 | MAP3K5 | HYDROXYUREA | unknown | PharmGKB |
| BAG6 | BAG6 | CARBAMAZEPINE | unknown | PharmGKB |
| HBEGF | HBEGF | CETUXIMAB | unknown | PharmGKB |
| HBEGF | HBEGF | PANITUMUMAB | unknown | PharmGKB |
| HSD17B10 | HSD17B10 | EPINEPHRINE BITARTRATE | unknown | DTC |
| HSD17B10 | HSD17B10 | SULFAPHENAZOLE | unknown | DTC |
| HSD17B10 | HSD17B10 | PSEUDOEPHEDRINE | unknown | DTC |
| HSD17B10 | HSD17B10 | TELMISARTAN | unknown | DTC |
| HSD17B10 | HSD17B10 | METHOTREXATE | unknown | DTC |
| HSD17B10 | HSD17B10 | CARBARIL | unknown | DTC |
| HSD17B10 | HSD17B10 | THIMEROSAL | unknown | DTC |
| HSD17B10 | HSD17B10 | HYDROCORTISONE | unknown | DTC |
| HSD17B10 | HSD17B10 | BUMETANIDE | unknown | DTC |
| HSD17B10 | HSD17B10 | AMSACRINE | unknown | DTC |
| HSD17B10 | HSD17B10 | LEVODOPA | unknown | DTC |
| HSD17B10 | HSD17B10 | HALOTHANE | unknown | DTC |
| HSD17B10 | HSD17B10 | RALOXIFENE | unknown | DTC |
| HSD17B10 | HSD17B10 | PROTRIPTYLINE | unknown | DTC |
| HSD17B10 | HSD17B10 | DOPAMINE | unknown | DTC |
| HSD17B10 | HSD17B10 | PREDNISONE | unknown | DTC |
| HSD17B10 | HSD17B10 | CIPROFLOXACIN | unknown | DTC |
| HSD17B10 | HSD17B10 | MALATHION | unknown | DTC |
| HSD17B10 | HSD17B10 | METHYLDOPA | unknown | DTC |
| HSD17B10 | HSD17B10 | NOREPINEPHRINE BITARTRATE | unknown | DTC |
| HSD17B10 | HSD17B10 | METOLAZONE | unknown | DTC |
| HSD17B10 | HSD17B10 | HYDROXYZINE PAMOATE | unknown | DTC |
| HSD17B10 | HSD17B10 | AMPHOTERICIN B | unknown | DTC |
| HSD17B10 | HSD17B10 | PRAZOSIN | unknown | DTC |
| HSD17B10 | HSD17B10 | HEXACHLOROPHENE | unknown | DTC |
| HSD17B10 | HSD17B10 | OXYTETRACYCLINE HYDROCHLORIDE | unknown | DTC |
| HSD17B10 | HSD17B10 | TRAZODONE | unknown | DTC |
| HSD17B10 | HSD17B10 | RIBOFLAVIN | unknown | DTC |
| HSD17B10 | HSD17B10 | MYCOPHENOLIC ACID | unknown | DTC |
| HSD17B10 | HSD17B10 | BENZBROMARONE | unknown | DTC |
| HSD17B10 | HSD17B10 | CISPLATIN | unknown | DTC |
| HSD17B10 | HSD17B10 | LABETALOL HYDROCHLORIDE | unknown | DTC |
| HSD17B10 | HSD17B10 | GRISEOFULVIN | unknown | DTC |
| HSD17B10 | HSD17B10 | LEVONORDEFRIN | unknown | DTC |
| HSD17B10 | HSD17B10 | ZOLPIDEM | unknown | DTC |
| HSD17B10 | HSD17B10 | FOLIC ACID | unknown | DTC |
| HSD17B10 | HSD17B10 | DIFLUNISAL | unknown | DTC |
| HSD17B10 | HSD17B10 | RALOXIFENE HYDROCHLORIDE | unknown | DTC |
| HSD17B10 | HSD17B10 | INAMRINONE | unknown | DTC |
| HSD17B10 | HSD17B10 | CARBOPLATIN | unknown | DTC |
| HSD17B10 | HSD17B10 | ALFUZOSIN | unknown | DTC |
| HSD17B10 | HSD17B10 | OFLOXACIN | unknown | DTC |
| HSD17B10 | HSD17B10 | ERGONOVINE | unknown | DTC |
| HSD17B10 | HSD17B10 | MESALAMINE | unknown | DTC |
| HSD17B10 | HSD17B10 | DEQUALINIUM | unknown | DTC |
| HSD17B10 | HSD17B10 | PRAZOSIN HYDROCHLORIDE | unknown | DTC |
| HSD17B10 | HSD17B10 | NIFEDIPINE | unknown | DTC |
| HSD17B10 | HSD17B10 | EPINEPHRINE | unknown | DTC |
| HSD17B10 | HSD17B10 | AMILORIDE HYDROCHLORIDE | unknown | DTC |
| HSD17B10 | HSD17B10 | AMILORIDE | unknown | DTC |
| HSD17B10 | HSD17B10 | PIRFENIDONE | unknown | DTC |
| HSD17B10 | HSD17B10 | CARBIDOPA | unknown | DTC |
| HSD17B10 | HSD17B10 | PYROGALLOL | unknown | DTC |
| HSD17B10 | HSD17B10 | DISULFIRAM | unknown | DTC |
| HSD17B10 | HSD17B10 | GLIQUIDONE | unknown | DTC |
| HSD17B10 | HSD17B10 | PIRETANIDE | unknown | DTC |
| HSD17B10 | HSD17B10 | SALMETEROL XINAFOATE | unknown | DTC |
| HSD17B10 | HSD17B10 | TRAZODONE HYDROCHLORIDE | unknown | DTC |
| HSD17B10 | HSD17B10 | ACRISORCIN | unknown | DTC |
| HSD17B10 | HSD17B10 | TRIAMTERENE | unknown | DTC |
| HSD17B10 | HSD17B10 | ETHAMSYLATE | unknown | DTC |
| HSD17B10 | HSD17B10 | HYDROQUINONE | unknown | DTC |
| HSD17B10 | HSD17B10 | APOMORPHINE | unknown | DTC |
| HSD17B10 | HSD17B10 | PADIMATE O | unknown | DTC |
| HSD17B10 | HSD17B10 | DIPYRIDAMOLE | unknown | DTC |
| PSMD4 | PSMD4 | TALAZOPARIB | unknown | CIViC |
| PTAFR | PTAFR | TICLOPIDINE | unknown | TTD |
| HLA-DQB1 | HLA-DQB1 | TICLOPIDINE | unknown | PharmGKB |
| HLA-DQB1 | HLA-DQB1 | CLAVULANIC ACID | unknown | PharmGKB |
| HLA-DQB1 | HLA-DQB1 | NEVIRAPINE | unknown | PharmGKB |
| HLA-DQB1 | HLA-DQB1 | AMOXICILLIN | unknown | PharmGKB |
| HLA-DQB1 | HLA-DQB1 | LAMOTRIGINE | unknown | PharmGKB |
| HLA-DQB1 | HLA-DQB1 | ASPIRIN | unknown | PharmGKB |
| HLA-DQB1 | HLA-DQB1 | FLOXACILLIN | unknown | PharmGKB |
| HLA-DQB1 | HLA-DQB1 | CARBAMAZEPINE | unknown | PharmGKB |
| HLA-DQB1 | HLA-DQB1 | ACETAMINOPHEN | unknown | PharmGKB |
| IL2RG | IL2RG | DENILEUKIN DIFTITOX | unknown | ChemblInteractions |
| XBP1 | XBP1 | FLUSPIRILENE | unknown | DTC |
| XBP1 | XBP1 | PIMOZIDE | unknown | DTC |
| IL6R | IL6R | BAZEDOXIFENE | unknown | DTC |
| IL6R | IL6R | RALOXIFENE | unknown | DTC |
| IL6R | IL6R | THALIDOMIDE | unknown | NCI |
| IL6R | IL6R | FLUOROURACIL | unknown | NCI |
| MDH2 | MDH2 | CISPLATIN | unknown | NCI |
| MDH2 | MDH2 | ALBUMIN HUMAN | unknown | NCI |
| FCGR3B | FCGR3B | PROGESTERONE | unknown | NCI |
| FCGR3B | FCGR3B | SODIUM CHLORIDE | unknown | NCI |
| FCGR3B | FCGR3B | CYCLOSPORINE | unknown | NCI |
| FCGR3B | FCGR3B | INDOMETHACIN | unknown | NCI |
| FCGR3B | FCGR3B | METHOTREXATE | unknown | NCI |
| FCGR3B | FCGR3B | PREDNISOLONE | unknown | NCI |
| FCGR3B | FCGR3B | THALIDOMIDE | unknown | NCI |
| FCGR3B | FCGR3B | CHOLECALCIFEROL | unknown | NCI |
| FCGR3B | FCGR3B | EPOETIN ALFA | unknown | NCI |
| FCGR3B | FCGR3B | PENICILLIN G POTASSIUM | unknown | NCI |
| FCGR3B | FCGR3B | METHIMAZOLE | unknown | NCI |
| FCGR3B | FCGR3B | LACTULOSE | unknown | NCI |
| FCGR3B | FCGR3B | FENTANYL | unknown | NCI |
| FCGR3B | FCGR3B | DOXORUBICIN | unknown | NCI |
| SYK | SYK | IMATINIB MESYLATE | unknown | TTD |
| SYK | SYK | DASATINIB | unknown | DTC |
| SYK | SYK | ERLOTINIB | unknown | DTC |
| SYK | SYK | PACLITAXEL | unknown | CIViC |
| UBE2N | UBE2N | PERPHENAZINE | unknown | DTC |
| UBE2N | UBE2N | RALOXIFENE HYDROCHLORIDE | unknown | DTC |
| UBE2N | UBE2N | THONZONIUM BROMIDE | unknown | DTC |
| ARF6 | ARF6 | INSULIN | unknown | NCI |
| ARID1A | ARID1A | NIVOLUMAB | unknown | CIViC |
| ARID1A | ARID1A | PEMBROLIZUMAB | unknown | CIViC |
| ARID1A | ARID1A | ATEZOLIZUMAB | unknown | CIViC |
| ARID1A | ARID1A | SORAFENIB | unknown | CIViC |
| ARID1A | ARID1A | DASATINIB | unknown | CIViC |
| CD55 | CD55 | ALCOHOL | unknown | NCI |
| YWHAE | YWHAE | INSULIN | unknown | NCI |
| PRKCD | PRKCD | ALCOHOL | unknown | NCI |
| CTDSP1 | CTDSP1 | ISOPROPAMIDE IODIDE | unknown | DTC |
| CTDSP1 | CTDSP1 | CEFUROXIME SODIUM | unknown | DTC |
| CTDSP1 | CTDSP1 | CEPHALOTHIN | unknown | DTC |
| CTDSP1 | CTDSP1 | CEFACLOR | unknown | DTC |
| CTDSP1 | CTDSP1 | CEFDINIR | unknown | DTC |
| RBL2 | RBL2 | SIROLIMUS | unknown | NCI |
| WRB | GET1 | METHYLPHENIDATE | unknown | PharmGKB |
| STAT6 | STAT6 | DOXORUBICIN HYDROCHLORIDE | unknown | DTC |
| STAT6 | STAT6 | INDOMETHACIN | unknown | NCI |
| CAST | CAST | THROMBIN | unknown | NCI |
| CAST | CAST | EPINEPHRINE | unknown | NCI |
| CAPG | CAPG | VINCRISTINE | unknown | PharmGKB |
| RXRA | RXRA | TRICLOSAN | unknown | DTC |
| RXRA | RXRA | RALOXIFENE HYDROCHLORIDE | unknown | DTC |
| RXRA | RXRA | DOXORUBICIN HYDROCHLORIDE | unknown | DTC |
| RXRA | RXRA | DOCETAXEL | unknown | PharmGKB |
| RXRA | RXRA | PHENOXYETHANOL | unknown | DTC |
| RXRA | RXRA | PROGESTERONE | unknown | DTC |
| RXRA | RXRA | IMIPRAMINE HYDROCHLORIDE | unknown | DTC |
| RXRA | RXRA | THIMEROSAL | unknown | DTC |
| RXRA | RXRA | TRETINOIN | unknown | DTC |
| RXRA | RXRA | DAUNORUBICIN HYDROCHLORIDE | unknown | DTC |
| RXRA | RXRA | DACTINOMYCIN | unknown | DTC |
| RXRA | RXRA | TRICLOCARBAN | unknown | DTC |
| RXRA | RXRA | NICLOSAMIDE | unknown | DTC |
| RXRA | RXRA | PIMOZIDE | unknown | DTC |
| RXRA | RXRA | SAQUINAVIR MESYLATE | unknown | DTC |
| RXRA | RXRA | TESTOSTERONE | unknown | DTC |
| HDAC1 | HDAC1 | DAUNORUBICIN | unknown | DTC |
| HDAC1 | HDAC1 | VALPROIC ACID | unknown | DTC |
| HDAC1 | HDAC1 | PHENYLBUTANOIC ACID | unknown | DTC|TTD |
| SUMO4 | SUMO4 | TACROLIMUS | unknown | PharmGKB |
| HSP90AB1 | HSP90AB1 | DIPYRIDAMOLE | unknown | DTC |
| HSP90AB1 | HSP90AB1 | DIACEREIN | unknown | DTC |
| HSP90AB1 | HSP90AB1 | DAUNORUBICIN HYDROCHLORIDE | unknown | DTC |
| HSP90AB1 | HSP90AB1 | BEVACIZUMAB | unknown | PharmGKB |
| HSP90AB1 | HSP90AB1 | DOXORUBICIN HYDROCHLORIDE | unknown | DTC |
| CXCR4 | CXCR4 | BEVACIZUMAB | unknown | PharmGKB |
| CXCR4 | CXCR4 | CISPLATIN | unknown | CIViC |
| PKM | PKM | HYDRALAZINE | unknown | DTC |
| PKM | PKM | SULFADIAZINE, SILVER | unknown | DTC |
| PKM | PKM | NICLOSAMIDE | unknown | DTC |
| PKM | PKM | PSEUDOEPHEDRINE | unknown | DTC |
| PKM | PKM | NITAZOXANIDE | unknown | DTC |
| PKM | PKM | AMLEXANOX | unknown | DTC |
| CDC42 | CDC42 | GONADORELIN ACETATE | unknown | NCI |
| RAB27A | RAB27A | EMAPALUMAB | unknown | PharmGKB|FDA |
| FCER1A | FCER1A | DESLORATADINE | unknown | PharmGKB |
| FCER1A | FCER1A | MIZOLASTINE | unknown | PharmGKB |
| TOP2B | TOP2B | ROSOXACIN | unknown | TTD |
| TOP2B | TOP2B | DEXRAZOXANE | unknown | PharmGKB|TTD |
| TOP2B | TOP2B | MITOXANTRONE | unknown | TTD |
| TOP2B | TOP2B | VALRUBICIN | unknown | TTD |
| TOP2B | TOP2B | CINOXACIN | unknown | TTD |
| TOP2B | TOP2B | DIGITOXIN | unknown | DTC |
| TOP2B | TOP2B | LOMEFLOXACIN | unknown | TTD |
| TOP2B | TOP2B | DOXORUBICIN | unknown | ClearityFoundationClinicalTrial|PharmGKB|TTD |
| TOP2B | TOP2B | AMSACRINE | unknown | TTD |
| TOP2B | TOP2B | ENOXACIN | unknown | TTD |
| TOP2B | TOP2B | IDARUBICIN | unknown | TTD |
| TOP2B | TOP2B | PEFLOXACIN | unknown | TTD |
| TOP2B | TOP2B | EPIRUBICIN | unknown | TTD |
| TOP2B | TOP2B | PODOFILOX | unknown | TTD |
| TOP2B | TOP2B | PIXANTRONE | unknown | TTD |
| P4HB | P4HB | RALOXIFENE HYDROCHLORIDE | unknown | DTC |
| GZMB | GZMB | HEXACHLOROPHENE | unknown | DTC |
| FKBP5 | FKBP5 | NEFAZODONE | unknown | PharmGKB |
| FKBP5 | FKBP5 | CLOZAPINE | unknown | PharmGKB |
| FKBP5 | FKBP5 | VENLAFAXINE | unknown | PharmGKB |
| FKBP5 | FKBP5 | PAROXETINE | unknown | PharmGKB |
| FKBP5 | FKBP5 | CITALOPRAM | unknown | PharmGKB |
| FKBP5 | FKBP5 | BUPROPION | unknown | PharmGKB |
| FKBP5 | FKBP5 | CLOMIPRAMINE | unknown | PharmGKB |
| FKBP5 | FKBP5 | FLUOXETINE | unknown | PharmGKB |
| FKBP5 | FKBP5 | GEMCITABINE | unknown | PharmGKB |
| FKBP5 | FKBP5 | ESCITALOPRAM | unknown | PharmGKB |
| FKBP5 | FKBP5 | MIRTAZAPINE | unknown | PharmGKB |
| HINT1 | HINT1 | NICOTINE | unknown | PharmGKB |
| CSK | CSK | HYDROCHLOROTHIAZIDE | unknown | PharmGKB |
| ITGAL | ITGAL | FLUOROURACIL | unknown | NCI |
| ITGAL | ITGAL | EPOETIN ALFA | unknown | NCI |
| ITGAL | ITGAL | ETOPOSIDE | unknown | NCI |
| ITGAL | ITGAL | THROMBIN | unknown | NCI |
| ITGAL | ITGAL | MYCOPHENOLATE MOFETIL | unknown | NCI |
| ITGAL | ITGAL | CYCLOSPORINE | unknown | NCI |
| ITGAL | ITGAL | SIROLIMUS | unknown | NCI |
| ITGAL | ITGAL | CYCLOPHOSPHAMIDE | unknown | NCI |
| ITGAL | ITGAL | BUSULFAN | unknown | NCI |
| VASP | VASP | HYDROCHLOROTHIAZIDE | unknown | PharmGKB |
| RAC1 | RAC1 | DABRAFENIB | unknown | CIViC |
| RAC1 | RAC1 | VEMURAFENIB | unknown | CIViC |
| CCR2 | CCR2 | MORPHINE | unknown | NCI |
| CCR2 | CCR2 | PLERIXAFOR | unknown | DTC |
| CCR2 | CCR2 | SIMVASTATIN | unknown | NCI |
| FYN | FYN | VANDETANIB | unknown | DTC |
| FYN | FYN | PAZOPANIB | unknown | DTC |
| ARRB2 | ARRB2 | FENTANYL | unknown | PharmGKB |
| ARRB2 | ARRB2 | BUPRENORPHINE | unknown | PharmGKB |
| ARRB2 | ARRB2 | TRAMADOL | unknown | PharmGKB |
| ICAM3 | ICAM3 | CYCLOSPORINE | unknown | NCI |
| ICAM3 | ICAM3 | METHOTREXATE | unknown | NCI |
| GSTP1 | GSTP1 | ALCOHOL | unknown | NCI |
| GSTP1 | GSTP1 | DAUNORUBICIN | unknown | PharmGKB |
| GSTP1 | GSTP1 | LEUCOVORIN | unknown | PharmGKB |
| GSTP1 | GSTP1 | CYTARABINE | unknown | NCI |
| GSTP1 | GSTP1 | ISONIAZID | unknown | PharmGKB |
| GSTP1 | GSTP1 | BUSULFAN | unknown | NCI|PharmGKB |
| GSTP1 | GSTP1 | PREDNISONE | unknown | NCI |
| GSTP1 | GSTP1 | MELPHALAN | unknown | NCI |
| GSTP1 | GSTP1 | VERAPAMIL | unknown | NCI |
| GSTP1 | GSTP1 | THIOTEPA | unknown | PharmGKB |
| GSTP1 | GSTP1 | DECITABINE | unknown | NCI |
| GSTP1 | GSTP1 | IFOSFAMIDE | unknown | NCI |
| GSTP1 | GSTP1 | CISPLATIN | unknown | NCI|CIViC|PharmGKB |
| GSTP1 | GSTP1 | DEXAMETHASONE | unknown | NCI |
| GSTP1 | GSTP1 | AZACITIDINE | unknown | NCI |
| GSTP1 | GSTP1 | FLUOROURACIL | unknown | PharmGKB |
| GSTP1 | GSTP1 | RIFAMPIN | unknown | PharmGKB |
| GSTP1 | GSTP1 | EPIRUBICIN | unknown | PharmGKB |
| GSTP1 | GSTP1 | OMEPRAZOLE | unknown | NCI |
| GSTP1 | GSTP1 | OXALIPLATIN | unknown | NCI|PharmGKB |
| GSTP1 | GSTP1 | CARBOPLATIN | unknown | NCI|CIViC |
| GSTP1 | GSTP1 | DOXORUBICIN | unknown | PharmGKB |
| GSTP1 | GSTP1 | DOCETAXEL | unknown | NCI |
| GSTP1 | GSTP1 | PYRIMETHAMINE | unknown | PharmGKB |
| GSTP1 | GSTP1 | SELENOMETHIONINE | unknown | NCI |
| GSTP1 | GSTP1 | IRINOTECAN HYDROCHLORIDE | unknown | NCI |
| GSTP1 | GSTP1 | HYDROQUINONE | unknown | NCI |
| GSTP1 | GSTP1 | BLEOMYCIN | unknown | PharmGKB |
| GSTP1 | GSTP1 | CYCLOPHOSPHAMIDE | unknown | NCI|PharmGKB |
| GSTP1 | GSTP1 | PACLITAXEL | unknown | CIViC |
| GSTP1 | GSTP1 | ETOPOSIDE | unknown | PharmGKB |
| PGK1 | PGK1 | LAMIVUDINE | unknown | PharmGKB |
| PPA1 | PPA1 | ALCOHOL | unknown | NCI |
| CSF3R | CSF3R | PEXIDARTINIB | unknown | TTD |
| CSF3R | CSF3R | RUXOLITINIB | unknown | JAX-CKB|DoCM|CIViC |
| CYBA | CYBA | SIMVASTATIN | unknown | PharmGKB |
| CYBA | CYBA | DOXORUBICIN | unknown | PharmGKB |
| CYBA | CYBA | IDARUBICIN | unknown | PharmGKB |
| CST3 | CST3 | DIGOXIN | unknown | NCI |
| CST3 | CST3 | RIBAVIRIN | unknown | NCI |
| PTPN6 | PTPN6 | TOFACITINIB | unknown | DTC |
| PTPN6 | PTPN6 | SORAFENIB | unknown | DTC |
| CSF1R | CSF1R | PROGESTERONE | unknown | NCI |
| CSF1R | CSF1R | VEMURAFENIB | unknown | JAX-CKB |
| CSF1R | CSF1R | SORAFENIB | unknown | DTC|JAX-CKB |
| NPM1 | NPM1 | CRIZOTINIB | unknown | JAX-CKB |
| NPM1 | NPM1 | MIDOSTAURIN | unknown | PharmGKB|FDA |
| NPM1 | NPM1 | VENETOCLAX | unknown | PharmGKB|FDA |
| NPM1 | NPM1 | ALECTINIB | unknown | JAX-CKB |
| NPM1 | NPM1 | CERITINIB | unknown | JAX-CKB |
| NPM1 | NPM1 | TRETINOIN | unknown | CIViC |
| NPM1 | NPM1 | VORINOSTAT | unknown | JAX-CKB |
| NPM1 | NPM1 | IXAZOMIB | unknown | JAX-CKB |
| NPM1 | NPM1 | LORLATINIB | unknown | JAX-CKB |
| CLC | CLC | MOXIDECTIN | unknown | TTD |
| DPYSL2 | DPYSL2 | ERLOSAMIDE | unknown | TdgClinicalTrial|TTD |
| LST1 | LST1 | ABACAVIR | unknown | PharmGKB |
| ITGAM | ITGAM | HYDROCORTISONE | unknown | NCI |
| ITGAM | ITGAM | CLARITHROMYCIN | unknown | NCI |
| ITGAM | ITGAM | PHENYLEPHRINE | unknown | NCI |
| ITGAM | ITGAM | ATORVASTATIN | unknown | NCI |
| ITGAM | ITGAM | THEOPHYLLINE | unknown | NCI |
| ITGAM | ITGAM | DIMETHYL SULFOXIDE | unknown | NCI |
| ITGAM | ITGAM | MORPHINE | unknown | NCI |
| ITGAM | ITGAM | FENTANYL | unknown | NCI |
| UCP2 | UCP2 | SOYBEAN OIL | unknown | NCI |
| UCP2 | UCP2 | LIOTHYRONINE SODIUM | unknown | NCI |
| UCP2 | UCP2 | SODIUM CHLORIDE | unknown | NCI |
| CSF2RB | CSF2RB | TAGRAXOFUSP | unknown | TTD |
| RAC2 | RAC2 | DOXORUBICIN | unknown | PharmGKB |
| RAC2 | RAC2 | IDARUBICIN | unknown | PharmGKB |
| DUSP6 | DUSP6 | TRAMETINIB | unknown | CIViC |
| HLA-DPB1 | HLA-DPB1 | ASPIRIN | unknown | PharmGKB |
| HLA-DPB1 | HLA-DPB1 | CLOZAPINE | unknown | PharmGKB |
| TUBA1B | TUBA1B | VINORELBINE | unknown | DTC |
| TUBA1B | TUBA1B | VINCRISTINE | unknown | DTC |
| TUBA1B | TUBA1B | VORINOSTAT | unknown | DTC |
| TUBA1B | TUBA1B | VINBLASTINE | unknown | DTC |
| TUBA1B | TUBA1B | PODOFILOX | unknown | DTC |
| TUBA1C | TUBA1C | VINCRISTINE | unknown | DTC |
| TUBA1C | TUBA1C | VINBLASTINE | unknown | DTC |
| TUBA1C | TUBA1C | VORINOSTAT | unknown | DTC |
| TUBA1C | TUBA1C | PODOFILOX | unknown | DTC |
| TUBA1C | TUBA1C | VINORELBINE | unknown | DTC |
| HLA-G | HLA-G | FLUOROURACIL | unknown | PharmGKB |
| HLA-G | HLA-G | METHOTREXATE | unknown | PharmGKB |
| HLA-G | HLA-G | CAPECITABINE | unknown | PharmGKB |
| S100A12 | S100A12 | METHOTREXATE | unknown | NCI |
| S100A8 | S100A8 | METHOTREXATE | unknown | NCI |
| HLA-C | HLA-C | CLOZAPINE | unknown | PharmGKB |
| HLA-C | HLA-C | RIBAVIRIN | unknown | PharmGKB |
| HLA-C | HLA-C | GEMCITABINE | unknown | PharmGKB |
| HLA-C | HLA-C | AMOXICILLIN | unknown | PharmGKB |
| HLA-C | HLA-C | METHOTREXATE | unknown | PharmGKB |
| HLA-C | HLA-C | LAMOTRIGINE | unknown | PharmGKB |
| HLA-C | HLA-C | CARBOPLATIN | unknown | PharmGKB |
| HLA-C | HLA-C | CLAVULANIC ACID | unknown | PharmGKB |
| HLA-C | HLA-C | METHAZOLAMIDE | unknown | PharmGKB |
| HLA-C | HLA-C | USTEKINUMAB | unknown | PharmGKB |
| HLA-C | HLA-C | FLOXACILLIN | unknown | PharmGKB |
| HLA-C | HLA-C | TICLOPIDINE | unknown | PharmGKB |
